# Exploring the Scope of Macrocyclic “Shoe-last” Templates in the Mechanochemical Synthesis of RHO Topology Zeolitic Imidazolate Frameworks (ZIFs)

**DOI:** 10.3390/molecules25030633

**Published:** 2020-02-01

**Authors:** Ivana Brekalo, David E. Deliz, Christopher M. Kane, Tomislav Friščić, K. Travis Holman

**Affiliations:** 1 Department of Chemistry, Georgetown University, Washington, DC 20057, USA; ib308@georgetown.edu (I.B.); ded46@georgetown.edu (D.E.D.); chris.kane@angstrom.uu.se (C.M.K.); 2 Department of Chemistry, McGill University, Montreal, QC H3A 0B8, Canada

**Keywords:** mechanochemical synthesis, zeolitic imidazolate frameworks, templation, microporous materials

## Abstract

The macrocyclic cavitand **MeMeCH_2_** is used as a template for the mechanochemical synthesis of 0.2**MeMeCH_2_**@RHO-Zn_16_(**Cl_2_Im**)_32_ (0.2**MeMeCH_2_**@ZIF-71) and RHO-Zn**BIm**_2_ (ZIF-11) zeolitic imidazolate frameworks (ZIFs). It is shown that **MeMeCH_2_** significantly accelerates the mechanochemical synthesis, providing high porosity products (BET surface areas of 1140 m^2^/g and 869 m^2^/g, respectively). Templation of RHO-topology ZIF frameworks constructed of linkers larger than benzimidazole (HBIm) was unsuccessful. It is also shown that cavitands other than **MeMeCH_2_**—namely **MeHCH_2_**, **Me*i*BuCH_2_**, **HPhCH_2_**, **MePhCH_2_**, **BrPhCH_2_**, **BrC5CH_2_**—can serve as effective templates for the synthesis of *x*(cavitand)@RHO-ZnIm_2_ products. The limitations on cavitand size and shape are explored in terms of their effectiveness as templates.

## 1. Introduction

Zeolitic imidazolate frameworks (ZIFs) [1,2], zeolitic metal organic frameworks (ZMOFs) [3,4], tetrahedral imidazolate frameworks (TIFs) [5], or boron imidazolate frameworks (BIFs) [6] form a class of metal organic frameworks (MOFs) that can be considered as expanded structural analogues of zeolites. They are generally built from tetrahedral metal centers bridged with ditopic ligands/linkers, where the metal-linker-metal bonding angles mimic those found in zeolites, resulting in materials with extended 3D framework structures [7] analogous to zeolitic ones. Replacing the zeolitic bridging oxide ligands, and the Si/Al centers with tetrahedral transition metals, not only results in larger unit cell and pore sizes, but also has the added benefit of permitting easy functionalization and access to a range of ligand and metal combinations. Ligand identity can also affect framework flexibility and stability, as the ligand substituents can protect the metal from nucleophilic attack, or steric interactions between ligand substituents can form a “secondary network” that supports the framework architecture [8]. To date, over one hundred different metal-ligand ZIF compositions have been realized, adopting over sixty different tetrahedral framework structural (topological) variants [9].

The enormous structural diversity of ZIFs (and, more broadly, ZMOFs), however, inevitably leads to issues of synthetic reproducibility and challenges with topological control. Indeed, syntheses of ZIFs often yield a number of different topological forms (i.e., framework polymorphs, once emptied) from the same set of starting materials and similar reaction conditions. Different ZIF products often occur concomitantly within the same batch preparation. Moreover, under the reaction conditions, the product distributions can often evolve over time, further complicating the synthesis [10,11]. Consequently, potentially important ZIF materials have sometimes been observed only as individual single crystals in a preparation, and their syntheses can sometimes be very difficult to reproduce. For example, though the single crystal structure of the high porosity merlinoite (MER) topological form of zinc imidazolate (Zn**Im**_2_), ZIF10 or MER-Zn**Im**_2_, was reported in 2006 [2], the MER form of Zn**Im**_2_ was only prepared in phase-pure form, and fully characterized, ten years later [10]. These synthetic issues are especially prevalent for the less dense, larger pore ZIF topologies which are often not the most thermodynamically stable crystal form for a given metal-ligand combination [12]. As high-porosity ZIFs tend to be the most potentially important ones, the issue of framework topological control and reproducible synthesis is pressing.

Commonly, topological control in ZIF syntheses is achieved by developing successful synthetic recipes. Choice of metal [13], exploitation of ligand sterics [14,15], use of solvents as putative templates [16,17,18,19], choice of counterions (for charged ZMOFs) [3] and reaction conditions (e.g., reagents, base, temperature, time) [17], can all dramatically influence the topological outcome in ZIF syntheses_._ Novel framework topologies for certain metal-ligand combinations have also been achieved by post-synthetic linker or metal ion exchange [20,21]. Considering solvents, for example, ZIFs with high porosity have been achieved by using large amide solvents, such as dipropyl and dibutylformamide [19], which are included within the pores during synthesis. Several ligand-choice strategies have also been employed, as some ligands or metal-ligand combinations are known to favor certain topologies. For example, 2-nitroimidazole seems to bias mixed-ligand reactions toward the gmelinite (GME) topology [15], and 4,5-disubstituted imidazoles often provide RHO topology products [14]. Mixed ligand-steric index strategies have also been employed [22]. These approaches generally seek to limit the possible framework topological outcomes via sterics. Controlling the topological outcome within a family (where the ligand and metal are the same) of numerous possible framework polymorphs, however, can be significantly more challenging. A particularly challenging synthetic system is that of zinc imidazolate (Zn**Im**_2_), the simplest ZIF compositions. The unsubstituted imidazolate ligand is capable of conforming to the greatest number of possible framework topologies, as it is the least sterically demanding. Indeed, Zn**Im**_2_ has so far been prepared in 15 different crystalline topological forms that are stable at ambient pressure and temperature [23], as well as amorphous [24] and various temperature/pressure-dependent crystal forms [25]. Still, a large number of other topological forms are theoretically possible.

To tackle the problem of framework topological control and synthetic reproducibility in ZIFs, it seems reasonable to turn to templation, an approach often used in zeolite synthesis, and one that is gaining traction in the MOF community [26]. Unfortunately, unlike the negatively charged zeolite frameworks which can be templated by judicious choice of counter cations, most ZIFs are uncharged frameworks and therefore do not include counterions. It has become clear, however, that the C-H bonds of metal-coordinated imidazolate ligands are good hydrogen bond donors, akin to the C-H bonds of *N*,*N*-disubstituted imidazolium cations [27], which exhibit *pKa* values as low as 20 [28]. For example, the single crystal structures of many ZIF solvates reveal that the metal-coordinated imidazolate ligands engage C-H···acceptor hydrogen bonds with the included solvent (e.g., BCT-Zn**Im**_2_ (CSD reference codes VEJYIT, VEJYEP) [2,17] cag-Zn**Im**_2_ (VEJYUF) [2], CAN-Zn**Im**_2_ (PAJRUQ [19]). Also, the Farha group has explored the deprotonation of the acidic imidazolate hydrogen atom(s) in ZIFs such as SALEM-2 (SOD-Zn(**Im**)_1.7_(**mIm**)_0.3_, **mIm** = 2-methylimidazolate) [29]. Thus, we hypothesized [10,23] that templation of specific topological motifs could be achieved by introducing rigid molecules that can serve as hydrogen bond acceptors for the metal-imidazolate hydrogen bond donors, and whose size and symmetry is compatible to that of the desired motif.

**Figure 1:**
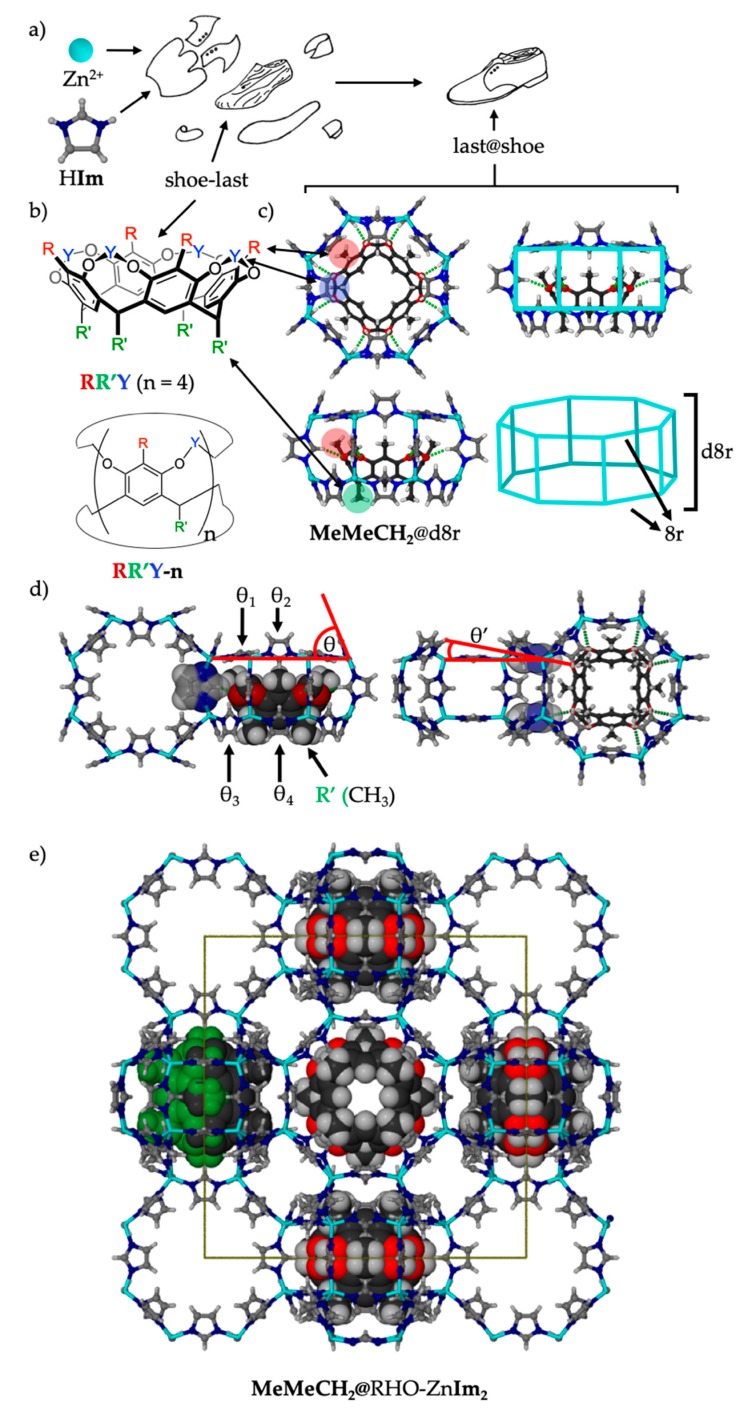
(**a**) Schematic representation of the “shoe-last” templating approach. (**b**) Cavitand naming scheme illustrating the *rccc* forms of RR’Y-*n* (where omitted, *n* = 4). (**c**) Top-down and side views of the general **MeMeCH_2_@***d8r* motif. **MeMeCH_2_** is bound *via* eight C-H···O hydrogen bonds (green dashes). (**d**) Crystal structure of **MeMeCH_2_**@RHO-Zn_16_(**Im**)_32_∙solvent, illustrating the fused empty and occupied *d8r*s with the empty *d8r* shown from the top and filled from the side (left) and vice-versa (right). Relevant θ angles of different *8r* imidazoles are pointed out. (**e**) The crystal structure of **MeMeCH_2_**@RHO-Zn**Im**_2_⋅*x*(solvent) as a ball-and-stick representation. Half of the *d8r*s are occupied by a (disordered) **MeMeCH_2_** cavitand, shown in spacefill. The disorder is represented by green and black color on one of the cavitand molecules.

Along these lines, we and others [30,31] have begun to explore the confinement of useful solute molecules within ZIFs, and it was reasoned that Cram’s ubiquitous and persistently bowl-shaped cavitands (**RR′Y**, Figure 1) [32] would be interesting candidates. Conveniently, cavitands can be easily modified at the upper (R) rims, lower (R′) rims, and bridging moieties (Y) of the bowl. They are available in various stereoisomeric forms related to the relative *cis/trans* stereochemistry of lower rim R’ substituents (*rccc* form depicted in Figure 1). They are also available in different ring sizes (e.g., *n* = 4, 5, 6, etc.), and many forms can be prepared in multigram quantities. Moreover, these stable molecules can typically withstand the conditions of ZIF syntheses. In exploring the possible encapsulation of cavitands by ZIFs, it was quickly discovered that one such cavitand, *rccc*-**MeMeCH_2_** (R = Me, R’ = Me, Y = CH_2_; with all methyl groups directed parallel to the *C*_4_-axis; hereafter, **MeMeCH_2_**), introduced into a solvothermal synthesis of zinc imidazolate, served to template the elusive MER topology of zinc imidazolate, as **MeMeCH_2_**@MER-Zn**Im**_2_, allowing, for the first time, synthesis of phase-pure material and characterization of its porosity [10]. Single crystal X-ray structural analysis of **MeMeCH_2_**@MER-ZnIm_2_⋅*x*(solvent) revealed the **MeMeCH_2_** template to be encapsulated within the double-8-ring (*d8r*) motifs of the MER framework. The eight short C-H···O hydrogen bonds (C···O = 3.135(6) Å, Figure 1) between the C-H groups at the 2-position of the imidazolate ligands of the Zn**Im**_2_ framework and the ether oxygen atoms of the encapsulated **MeMeCH_2_** cavitand clearly illustrate the role of the **MeMeCH_2_** as an eight-fold hydrogen bond acceptor template for the *d8r* motif. Moreover, the **MeMeCH_2_** template was found to be bound tightly within **MeMeCH_2_**@MER-Zn**Im**_2_, and was not susceptible to removal *via* solvent washing or thermal activation for sorption analysis. We therefore hypothesized that **MeMeCH_2_** acts as a “shoe-last” template around which the *d8r* motif (the “shoe”) is assembled.

This proof of concept result prompted us to explore the scope of the templation approach. Specifically, we wondered if we can induce the formation of other framework topologies, such as the RHO, TSC, PAU, and/or SBE zeolitic topologies, that exhibit the same *d8r* structural motif. Furthermore, we wanted to improve the templated synthesis, increasing yields, decreasing reaction times, and minimizing waste and energy consumption. To achieve these goals, we explored for the first time the mechanochemical templation of ZIF syntheses using a non-ionic solid (NIS) as template.

Mechanochemical synthesis of metal-organic frameworks in general has recently gained considerable attention [33,34,35]. For example, UiO-66 can be easily synthesized from simple zirconium benzoate or methacrylate precursors [36], and MOF-74 (or CPO-27), one of the quintessential MOFs, can be made from zinc oxide without bulk liquid [37]. Additionally, mechanochemical syntheses use almost no liquid, require little energy input (no heat), and often give quantitative yields in shorter times than solution based synthetic methods, making mechanochemistry a promising technique in the synthesis of MOFs [38]. Importantly, mechanochemistry has been applied to the synthesis of ZIFs, revealing that the direct reaction of metal oxides (ZnO/CoO) [39,40,41,42,43] and carbonates ([Zn(OH)_2_]_3_[ZnCO_3_]_2_) [44] with simple imidazoles can provide different ZIF materials under various mechanochemical conditions, including neat grinding (NG, milling without addition of liquid), liquid-assisted grinding (LAG, milling with the addition of catalytic amounts of liquid), ion-and-liquid-assisted grinding (ILAG, milling with the addition of catalytic amounts of liquid and simple inorganic salts), or accelerated aging (short milling followed by aging in liquid vapors, often warm water vapor).

Our recent combination of the “shoe-last” templation approach with mechanochemical synthesis resulted in the first synthesis of the highly porous RHO topological form of zinc imidazolate [23]. We demonstrated that short (2 min) milling of nanoparticulate zinc oxide, imidazole (H**Im**), and the **MeMeCH_2_** cavitand template with diethylformamide (DEF) in a molar ratio of 1:2:0.5:4, followed by aging of the resulting paste at ambient conditions in a capped vial (the LAG-aging procedure) results in essentially quantitative preparation of 0.9**MeMeCH_2_**@RHO-Zn_16_**Im**_32_. Single crystal X-ray structural analysis of a fortuitous crystal of **MeMeCH_2_**@RHO-Zn_16_**Im**_32_⋅*x*(solvent) grown *via* a solution-based synthesis showed that, as with the aforementioned **MeMeCH_2_**@MER-Zn_16_**Im**_32_⋅*x*(solvent) structure, the **MeMeCH_2_** template molecules occupy the *d8r* motifs (now inside the RHO framework, Figure 1d), and are engaged in eight short C-H···O contacts (C···O = 3.24(1) Å, Figure 1) with the framework imidazolates. The role of **MeMeCH_2_** as a hydrogen bond acceptor template was confirmed by the observation that the RHO topology product is not formed if the template is not used in mechanochemical synthesis, and removing the C-H hydrogen bond donor by using 2-methylimidazole instead of imidazole results in no RHO product. The formed 0.9**MeMeCH_2_**@RHO-Zn_16_**Im**_32_ is thermally stable up to >350 °C, highly porous (BET_N2,77K_ = 1650 m^2^/g), and can be synthesized in multigram quantities from inexpensive starting materials, using a solventless procedure with little energy input.

**Figure 2:**
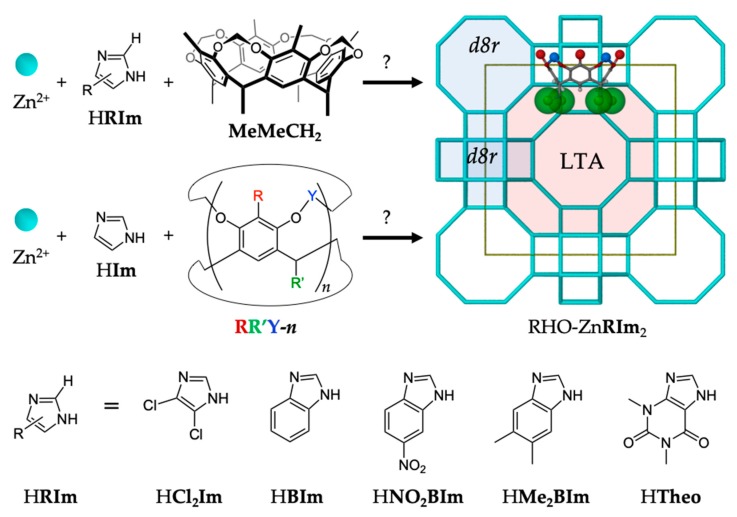
Exploration of the scope of the templation of the RHO topological form of zinc imidazolates, using different cavitands (see Table 1) and ligands.

We herein probe the limits of the RHO-topology ZIF templation effect by exploring different cavitand templates within the ZnO/H**Im** mechanochemical synthetic system. Conversely, we also explore the use of the **MeMeCH_2_** template for the templation of RHO-topological forms of ZIFs with different imidazolate ligands (**RIm**), as depicted in Figure 2. We proposed that structure-direction depends on the template fitting into the Zn**RIm**_2_ *d8r* motif (or at least a portion of it) and forming hydrogen bonds with the imidazolate moieties that make up the *d8r*. Therefore, the size/functionality of the imidazolate ligands and the size/shape of the putative cavitand templates were expected to have a substantial effect on the templation. In fact, adding/changing substituents on the imidazolate ligand is generally expected to limit the available topological landscape for a given ZIF synthesis. In this work we demonstrate the successful mechanochemical templation of RHO topological forms of zinc 4,5-dichloroimidazolate, RHO-Zn(**Cl_2_Im)**_2_ (ZIF-71 [14]), and the elusive zinc benzimidazolate, RHO-Zn**BIm**_2_ (ZIF-11 [2]), by the **MeMeCH_2_** cavitand, and the use of several different cavitands to prepare RHO-Zn**Im**_2_.

## 2. Results and Discussion

### 2.1. Accelerated Synthesis of RHO-Zn_16_(Cl_2_Im)_32_ and RHO-ZnBIm_2_ Using the MeMeCH_2_ Template

We previously demonstrated that the **MeMeCH_2_** template is unable to template RHO-topology ZIFs constructed with 2-substituted imidazolates (e.g., **2-Mem**), as the ligand cannot hydrogen bond with the template within a putative *d8r* system. Furthermore, to make any ZIF, both nitrogen atoms of the imidazole used must be non-substituted, as the nitrogen atoms bind to the zinc metal ions. Therefore, only ZIFs constructed with 5- and/or 4-substituted imidazolate ligands could be susceptible to RHO-templation by the **MeMeCH_2_** template.

**Figure 3:**
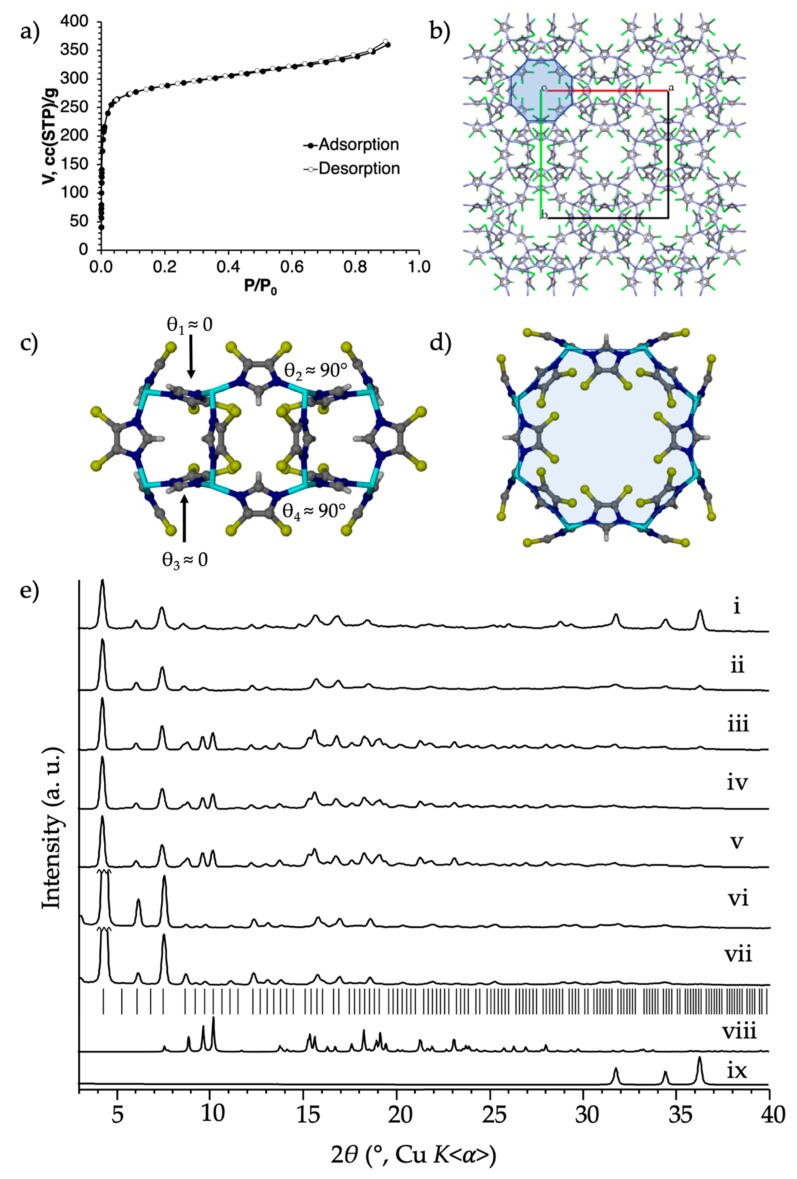
Synthesis and characterization of 0.2**MeMeCH_2_**@RHO-Zn_16_(**Cl_2_Im**)_32_ (0.2**MeMeCH_2_**@ZIF-71). (**a**) The 77 K low pressure N_2_ isotherm. (**b**) The crystal structure of RHO-Zn(**Cl_2_Im**)_2_ (CSD code GITVIP) as viewed down the [001] axis (the *d8r* motif is shaded blue). (**c**,**d**) Side and top views of the *d8r* motif of RHO-Zn(**Cl_2_Im**)_2_ (shaded blue). (**e**) Powder X-Ray diffraction (PXRD) patterns of the non-templated liquid-assisted grinding (LAG)-aging reaction of ZnO and H**Cl_2_Im** after (i) 3 days and (ii) 12 days; the **MeMeCH_2_** templated LAG-aging reaction of ZnO and H**Cl_2_Im** after (iii) 1 day and (iv) 3 days; the templated large scale (≈1.7 g) LAG-aging reaction of ZnO and H**Cl_2_Im** (v) after 3 days, and (vi) the washed 0.2**MeMeCH_2_**@RHO-Zn_16_(**Cl_2_Im**)_32_ final product after 4 days, and (vii) the same material after activation and 77 K low pressure N_2_ sorption analysis. (viii) Simulated PXRD pattern of **MeMeCH_2_⋅**2DEF (ix) PXRD pattern of nanoparticulate ZnO. Tick marks denote predicted peak positions of the RHO-Zn(**Cl_2_Im**)_2_ (ZIF-71, GITVIP).

**Figure 4:**
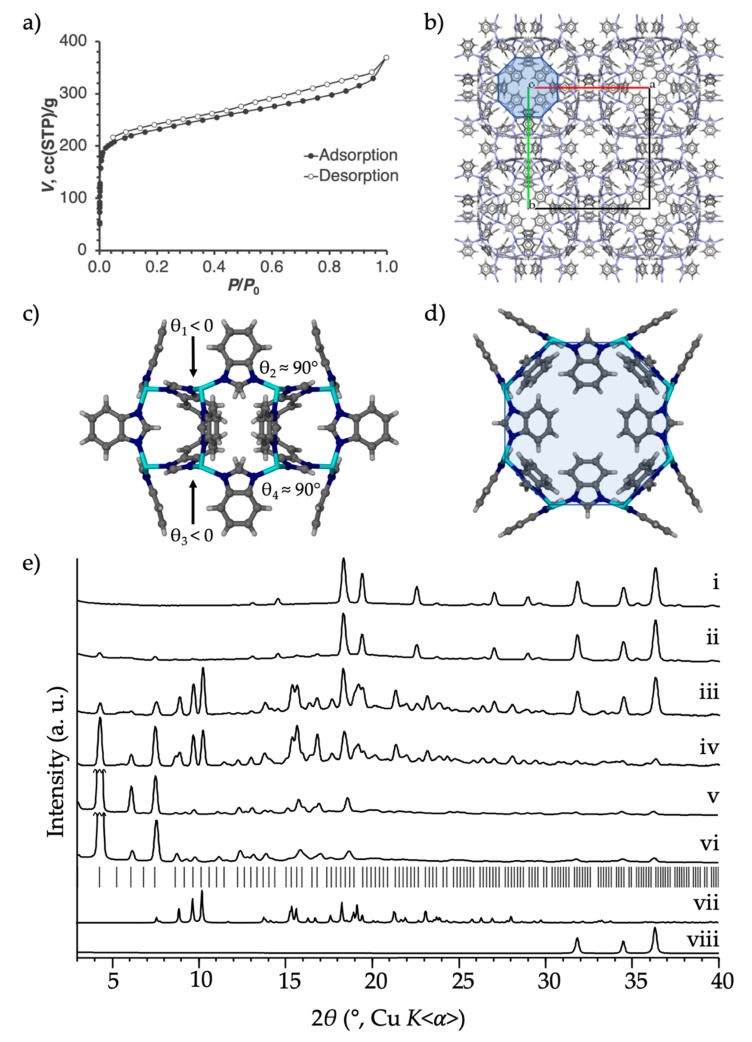
Synthesis and characterization of RHO-Zn**BIm**_2_ (ZIF-11). (**a**) The 77 K low pressure N_2_ isotherm. (**b**) The crystal structure of RHO-Zn**BIm**_2_ (CSD code VEJZOA) as viewed down the [001] axis (the *d8r* motif is shaded blue). (**c**,**d**) Side and top views of the d8r motif of RHO-Zn**BIm**_2_ (shaded blue). (**e**) PXRD patterns of the non-templated LAG-aging reaction of ZnO and H**BIm** after (i) 3 days, and (ii) 12 days; the **MeMeCH_2_** templated LAG-aging reaction of ZnO and H**BIm** after (iii) 3 days, (iv) 12 days, (v) the washed RHO-Zn**BIm**_2_ (ZIF-11) final product after 45 days, and (vi) the same material after activation and 77 K low pressure N_2_ sorption analysis. (vii) Simulated PXRD pattern of **MeMeCH_2_⋅**2DEF. (viii) PXRD pattern of nanoparticulate ZnO. Tick marks denote predicted peak positions of RHO-Zn**BIm**_2_ (ZIF-11, VEJZOA).

The first modification explored was the use of 4,5-dichloroimidazole, H**Cl_2_Im**. Two crystal forms of Zn(**Cl_2_Im**)_2_ are known to form *via* reaction of H**Cl_2_Im** with zinc salts (with no other ligands) in solution: RHO-Zn(**Cl_2_Im**)_2_ (ZIF-71), and **lcs**-Zn(**Cl_2_Im**)_2_ (ZIF-72) [14]. The RHO topology product (ZIF-71) is reportedly formed under higher dilution and higher temperature conditions, over a shorter period of time, and has since been used extensively as a sorbent [45,46]. It was therefore deemed interesting to determine whether RHO-Zn(**Cl_2_Im**)_2_ is amenable to our templation approach in mechanochemical synthesis. A liquid-assisted grinding (LAG)-aging synthesis was performed using nanoparticulate ZnO, H**Cl_2_Im**, and **MeMeCH_2_** as the templating agent, with DEF as the liquid additive. The reaction reached completion within approximately 4 days, and, after washing with chloroform to remove excess **MeMeCH_2_**, yielded essentially phase-pure 0.2**MeMeCH_2_**@RHO-Zn_16_(**Cl_2_Im**)_32_. The reaction conversion was nearly quantitative, based on powder X-ray diffraction (PXRD). NMR analysis suggests that only a tenth of the *d8r*-motifs (a fifth of accessible *d8r* motifs) in the RHO framework are filled with cavitand, a factor of about four less than the previously reported imidazolate-based material, 0.9**MeMeCH_2_**@RHO-Zn_16_**Im**_32_ [10]. The result suggests that increasing the ligand size may interfere with cavitand incorporation into the ZIF, likely due to steric effects. In the cavitand-free RHO ZIFs, the imidazolate ligands forming both 8-rings (*8r*) of the *d8r* motif alternate between being near parallel to the plane of the *8r* (θ ≤ 0, Figure 1c, Figure 3c and Figure 4c), and being near perpendicular to it (θ ≈ 90°, Figure 1c, Figure 3c and Figure 4c). For the cavitand template to fit into the *d8r*, imidazolates of the bottom *8r* must swing open so that they are all near parallel to the *d8r* axis (θ_3_ ≈ θ_4_ ≈ 90°, Figure 1c), while imidazolates of the top *8r* still alternate between being parallel and perpendicular (θ_1_ ≈ 0, θ_2_ ≈ 90°, Figure 1c). It is easy to predict that the all-perpendicular orientation will be harder to assume for ligands with increasing steric bulk.

Low temperature and pressure nitrogen sorption analysis of activated 0.2**MeMeCH_2_**@RHO-Zn_16_(**Cl_2_Im**)_32_ (Figure 3a) reveals a Type I isotherm and a surface area of 1140 m^2^/g. The material maintains crystallinity and its RHO topology after sorption, as evidenced by the post-sorption PXRD pattern. Interestingly, the surface area of 0.2**MeMeCH_2_**@RHO-Zn_16_(**Cl_2_Im**)_32_ is higher than the values previously reported for activated RHO-Zn_16_(**Cl_2_Im**)_32_ (ZIF-71: 652 m^2^/g [14] and 1050 m^2^/g [45]). The results suggest that the **MeMeCH_2_** cavitand may exhibit a stabilizing effect on the RHO-Zn_16_(**Cl_2_Im**)_32_ framework, potentially limiting framework flexibility with respect to contraction upon activation/emptying.

In a control reaction, the LAG-aging procedure was performed with the same metal source and ligand, nanoparticulate ZnO and H**Cl_2_Im**, but without the addition of the **MeMeCH_2_** template. Similar to the templated reaction, the non-templated control reaction also provides RHO-Zn(**Cl_2_Im**)_2_ (ZIF-71). However, monitoring the templated and non-templated reaction mixtures by PXRD over time revealed that the **MeMeCH_2_**-templated reaction is significantly faster. In fact, after only 1 day, the templated reaction shows approximately the same conversion (based on relative amount of ZnO) as the non-templated reaction does after 35 days. And though the non-templated reaction shows a large amount of residual ZnO after 3 days, the templated reaction undergoes full conversion in that time, both on a small (220 mg) and large (≈1.7 g) scale (Figure 3e). The results demonstrate that, despite the low level of incorporation of **MeMeCH_2_** into the RHO-Zn(**Cl_2_Im**)_2_ framework, not only does **MeMeCH_2_** help direct the topological outcome of a mechanochemical synthesis, it also allows the reaction to proceed significantly faster, and provides material of excellent porosity. Interestingly, this reaction is also significantly faster than the synthesis of 0.9**MeMeCH_2_**@RHO-Zn_16_**Im**_32_. We hypothesize that the increased acidity of H**Cl_2_Im** as compared to H**Im** is responsible for the increased reactivity and also stronger hydrogen-bonding interactions between the zinc-coordinated **Cl_2_Im^-^** ligands and the **MeMeCH_2_** template.

Encouraged by the successful templation of the RHO-Zn(**Cl_2_Im**)_2_ by the “shoe-last” template **MeMeCH_2_**, we turned to bulkier ligands. Benzimidazole was chosen as the next target ligand, seeking synthesis of RHO-Zn(**BIm**)_2_ (ZIF-11). The bulky ligands of ZIF-11 give rise to an unusual MOF structure; ZIF-11 possesses very large (~14.6 Å diameter) LTA (Linde Type A) cages, but very small apertures (<3 Å), making it of interest for the sieving of commodity gases, such as ethylene/ethane, CO_2_/H_2_, etc. [47]. Traditional solvothermal synthetic procedures have found that reaction of H**BIm** with tetrahedral metal ions gives only three ZIF topological outcomes in the absence of other co-ligands: the RHO topology RHO-Zn**BIm**_2_ (ZIF-11), RHO-Co**BIm**_2_ (ZIF-12) [2], the SOD topology SOD-Zn**BIm**_2_ (ZIF-7) [2], SOD-Co**BIm**_2_ (ZIF-9) [2], SOD-Cd**BIm**_2_ (CdIF-13) [48] or the layered, close-packed **sql**-Zn**BIm**_2_ [49]. **sql**-Zn**BIm**_2_ [42] and **sql**-Co**BIm**_2_ [50] have also been prepared by mechanochemical synthesis, by aging of a ZnO/H**BIm** mixture with addition of (NH_4_)_2_SO_4_ in H_2_O vapors, and LAG of a Co(NO_3_)_2_⋅6H_2_O/H**BIm** mixture with ethanol, respectively. Of the zinc-based materials, the SOD-Zn**BIm**_2_ and RHO-Zn**BIm**_2_ products are obtained under similar conditions, by the reaction of zinc nitrate tetrahydrate and benzimidazole *via* solvothermal methods. The SOD-Zn**BIm**_2_ (ZIF-7) product was obtained from DMF after heating at 130 °C for 2 days, and the RHO-Zn**BIm**_2_ (ZIF-11) product was obtained from DEF, under less concentrated conditions, and after 4 days of heating at 100 °C. A “toluene-assisted” synthesis of RHO-Zn**BIm**_2_ (ZIF-11) was later reported by He et al. using zinc acetate as the metal source, and ammonium hydroxide in ethanol as the solvent/base system [51]. Since this report (cited 50 times to date), the number of studies concerning the properties of RHO-Zn**BIm**_2_ (ZIF-11) have risen. In our hands, however, both the original and toluene-assisted procedures fail to give RHO-Zn**BIm**_2_ (see Figure S11). We note that others have synthesized RHO-Zn**BIm**_2_ (ZIF-11), citing He et al., but using a different solvent (e.g., MeOH [47]). It was hypothesized that our LAG-aging templation procedure might give a simple and reproducible way to synthesize this elusive member of the RHO family of zinc imidazolates.

A LAG-aging synthesis of RHO-Zn**BIm**_2_ (ZIF-11) was performed using nanoparticulate ZnO, H**BIm**, and **MeMeCH_2_** as the “shoe-last” template. The reaction proceeded significantly slower than the analogous H**Cl_2_Im** reaction; even after 12 days, a small amount of unreacted ZnO remained (based on PXRD analysis). The reaction, however, reached complete conversion within 45 days, and, after washing with chloroform to remove excess **MeMeCH_2_**, yielded phase-pure RHO-Zn**BIm**_2_ (ZIF-11, Figure 4e). ^1^H-NMR analysis of the digested RHO-Zn**BIm**_2_ product showed that, unlike 0.9**MeMeCH_2_**@RHO-Zn_16_(**Im**)_32_ and 0.2**MeMeCH_2_**@RHO-Zn_16_(**Cl_2_Im**)_32_, it contained no **MeMeCH_2_** residual template.

To confirm that, even though it is not incorporated into the product, **MeMeCH_2_** actually has a templation effect on the mechanochemical reaction, a control LAG-aging reaction was performed without **MeMeCH_2_**. The **MeMeCH_2_**-free control reaction provides very small amounts of RHO-Zn**BIm**_2_, after a much longer period of time than the **MeMeCH_2_**-templated reaction, as evidenced by PXRD (Figure 4e). Specifically, the **MeMeCH_2_**-free reaction shows no sign of RHO-Zn**BIm**_2_ product after 3 days, and only a very small amount after 12 days, with a very large amount of residual ZnO. The templated reaction, however, shows more conversion after 3 days than the **MeMeCH_2_**-free reaction after 12 days, and is nearly quantitative after 12 days (Figure 4e). The difference in reaction rate demonstrates that there is a templation effect: addition of **MeMeCH_2_** significantly accelerates the synthesis of the RHO-Zn**BIm**_2_ product. As mentioned, benzimidazole has so far been reported as a reagent in the mechanochemical synthesis of ZIFs only twice, using cobalt(II) nitrate or ZnO to synthesize the layered **sql**-topology Co**BIm**_2_ [50] or Zn**BIm**_2_ [42], respectively. This scarcity of reports is likely due to the very slow kinetics of the template-free reaction [42].

The question then remains: why is there no incorporation of the **MeMeCH_2_** template into the RHO-Zn**BIm**_2_ framework, if templation is happening? Earlier crystal structural analysis of **MeMeCH_2_**@RHO-Zn_16_(**Im**)_32_⋅*x*(solvent) (Figure 1) revealed that, due to the conformation of the imidazolates that must be assumed to allow C-H···O hydrogen bonding with the template in the **MeMeCH_2_**@*d8r* motif, all face-shared *d8r* neighbors must remain **MeMeCH_2_**-free, and a maximum of half of the *d8r*s (there are two *d8r*s per RHO-Zn_16_**RIm**_32_ formula unit) can be occupied by **MeMeCH_2_**.

It must be noted that, in the synthesis of bulk 0.9**MeMeCH_2_**@RHO-Zn_16_(**Im**)_32_, slightly less than half of the *d8r* motifs of the RHO frameworks are occupied by the **MeMeCH_2_** template. Regarding RHO-Zn**BIm**_2_, we hypothesize that, in the early stages of framework assembly the **MeMeCH_2_** may, *via* hydrogen bonding, transiently direct metal-coordinated imidazolate species into an orientation amenable to *d8r* and RHO formation — *via* a partially formed **MeMeCH_2_**@*d8r* motif — yet dissociate from those assemblies before formation of the **MeMeCH_2_**@*d8r* motif is complete. Indeed, incorporation of **MeMeCH_2_** into the *d8r* motif requires significant conformational reorientation of the imidazolate ligands within the 8-rings relative to the native empty *d8r* structure (see Figure 1 and Figure 2). Thus, we hypothesize that the greater steric bulk of the **BIm^-^** inhibits the necessary reorientation of the 8-ring ligands and thereby prevents formation of the completed, **MeMeCH_2_**-encapsulated **MeMeCH_2_**@*d8r* motifs. The template is thereby rejected from the material during later stages of *d8r*/RHO-framework formation, allowing it to be easily washed away in the chloroform rinsing step, leaving pure RHO-Zn**BIm**_2_. This hypothesis is also consistent with the observation that the degree of template incorporation in 0.2**MeMeCH_2_**@RHO-Zn_16_(**Cl_2_Im**)_32_ (derived from the moderately bulky Cl_2_Im^-^ ligand) lies between that of 0.9**MeMeCH_2_**@RHO-Zn_16_(**Im**)_32_ (non-bulky ligand) and **MeMeCH_2_**-templated RHO-Zn**BIm**_2_ (very bulky ligand).

Nitrogen sorption analysis of our sample of RHO-Zn**BIm**_2_ (ZIF-11, Figure 4a) gave surprising results. Whereas other samples of RHO-Zn**BIm**_2_ (ZIF-11) have, in multiple instances [2,52], been reported to be more or less “closed” to sorption of dinitrogen at 77 K—presumptively due to the small aperture sizes—our sample of RHO-Zn**BIm**_2_ exhibits a Type I isotherm with slight hysteresis in the mesoporous region, indicative of an open pore structure. The BET surface area measures 869 m^2^/g, and our RHO-Zn**BIm**_2_ material maintains crystallinity and the RHO topology after activation and sorption analysis, as evidenced by the post-sorption PXRD pattern (Figure 4e(vi)). Interestingly, though others’ samples of RHO-Zn**BIm**_2_ (ZIF-11) do not appear to be porous to dinitrogen at 77 K, the isostructural cobalt analog, RHO-Co**BIm**_2_ (ZIF-12), has been reported to readily absorb dinitrogen at 77 K [2,52]. The difference in behavior had previously been attributed to the slightly larger pore apertures in the cobalt-based material. We suggest that the unusual behavior of ZIF-11 may instead be due to flexibility of the RHO-Zn**BIm**_2_ framework. While traditional solvothermal synthesis provides ZIF-11 material with ligand conformations which block the pore opening (the *8r* motif, Figure 4c), it is possible that the association-dissociation of **MeMeCH_2_** during the early stages of RHO-Zn**BIm**_2_ framework assembly gives rise to a product with a more open conformation of the of **BIm**^-^ ligands in the 8-rings, as per the steric requirements of the cavitand described above. It is also possible that our RHO-Zn**BIm**_2_ is more defect-laden, providing avenues through which N_2_ can diffuse. Additionally, mechanochemical synthesis inherently provides materials with relatively small particle sizes [53], which increases the percentage of cages whose apertures are on the particle surface, where ring structures are inherently more flexible. This could potentially allow transient pore aperture widening and giving rise to a kinetic effect that allows observation of more rapid pore filling.

Finally, we attempted to synthesize RHO-Zn**RIm**_2_ frameworks with even bulkier ligands, in order to determine the limits of the templating procedure. We chose three benzimidazole derivatives with increasing numbers of substituents: 5-nitrobenzimidazole (H**NO_2_BIm**, one substituent), 5,6-dimethylbenzimidazole (H**Me_2_BIm**, two substituents), and theophylline (H**Theo**, four substituents). Of these, only one has been found to form a ZIF product without the addition of other ligands, namely **zea**-Zn(**Me_2_BIm**)_2_. Synthesis of **zea**-Zn(**Me_2_BIm**)_2_ has been achieved via solvothermal reaction of zinc acetate and H**Me_2_BIm** in 2-aminobut-1-ol and benzene at 150 °C [54]. It would therefore be very interesting if RHO topology ZIFs could be synthesized using these ligands by our approach. LAG-aging experiments were conducted by milling nanoparticulate ZnO, the **MeMeCH_2_** template and each of the ligands with DEF. Unfortunately, none of the three reactions gave any sign of an RHO topology product by PXRD, and only the H**Theo** reaction showed significant conversion to any product, giving the known discrete zinc theophylline dihydrate after about one year of aging (Figure S21).

We have therefore demonstrated that **MeMeCH_2_** templation of RHO-topology zinc imidazolate ZIFs in a mechanochemical reaction can be expanded to ligands smaller or equal in size to benzimidazole, with the amount of cavitand incorporation into the final RHO-Zn**RIm**_2_ product frameworks being inversely proportional to the size of the ligand.

### 2.2. Exploring Different Cavitand Templates in the Synthesis of (Template)_x_@RHO-ZnIm_2_ Materials

Having explored the ligand scope of the **MeMeCH_2_**-templated mechanochemical synthesis of RHO-Zn**RIm**_2_ ZIFs, we endeavored to explore the effect of template molecular structure on the topological outcome of the LAG-aging procedure using the simplest and least sterically demanding ligand, imidazole. The resulting (template)*_x_*@RHO-Zn**Im**_2_ materials are anticipated to have different properties—e.g., effective pore volume/diameter, framework flexibility, chemical and thermal stability—according to the level of incorporation and location of incorporation of the template and the size/shape or chemical functionality of the template. Moreover, RHO-Zn**Im**_2_ (with no template incorporation) remains unknown, and it would be of interest to compare the stability and properties of (template)*_x_*@RHO-Zn**Im**_2_ materials with native RHO-Zn**Im**_2_. Also, going forward, it would be of great interest to use the template@*d8r* encapsulation mechanism as a means to systematically introduce useful chemical functionalities (e.g., specific binding sites, catalytically active moieties, etc.) into the LTA cages of high porosity RHO-Zn**Im**_2_ ZIFs, for various applications. This could be done by chemically installing such moieties at the R’ “feet” of the encapsulated cavitand templates, which protrude into the large diameter (~1.5 nm) LTA cages of the ZIF. Thus, it was deemed useful to explore the steric limits of putative templates that can be encapsulated within (template)*_x_*@RHO-Zn**Im**_2_ materials.

It was originally hypothesized that the ideal template for a certain topological motif (here: the *d8r*) must match the motif in symmetry (4-fold or 8-fold for the *d8r*), shape (near cylindrical) and size, as well as have properly positioned hydrogen bond acceptors. To test this hypothesis we chose a larger cavitand of the “wrong” symmetry and total size (**MeHCH_2_**-6, *n* = 6, with 6 repeating resorcinol units, Figure 1), a cavitand of the “wrong” shape (**MeHSiMe_2_**, with an upper rim wider than the bottom rim and too wide for the *d8r*), and several cavitands with top (R = **H**, **Me, Br**) and bottom (R’ = **H**, **Me**, **Br**, ***iso*-butyl (*i*-Bu)**, **Ph**, 4-methylphenyl (**4-MePh**), and *n*-pentyl (**C_5_H_11_**)) rim substituents of varying sizes and lengths. Varying the R’ substituents was intended to explore the size/length limitations of substituents that can protrude into the LTA cages of the RHO-Zn**Im**_2_ frameworks. In addition to these cavitands, all of which conform to the *rccc* stereochemistry, a sample of **MePhCH_2_** cavitand containing 87% of the *rctt* stereoisomer—with two adjacent phenyl rings pointing orthogonal to the *C*_4_-axis of the cavitand bowl—was also explored. The *rctt-***MePhCH_2_** stereoisomer is mismatched to the *d8r*s in both size/shape (far too “wide” for the *d8r*) and overall symmetry.

**Figure 5:**
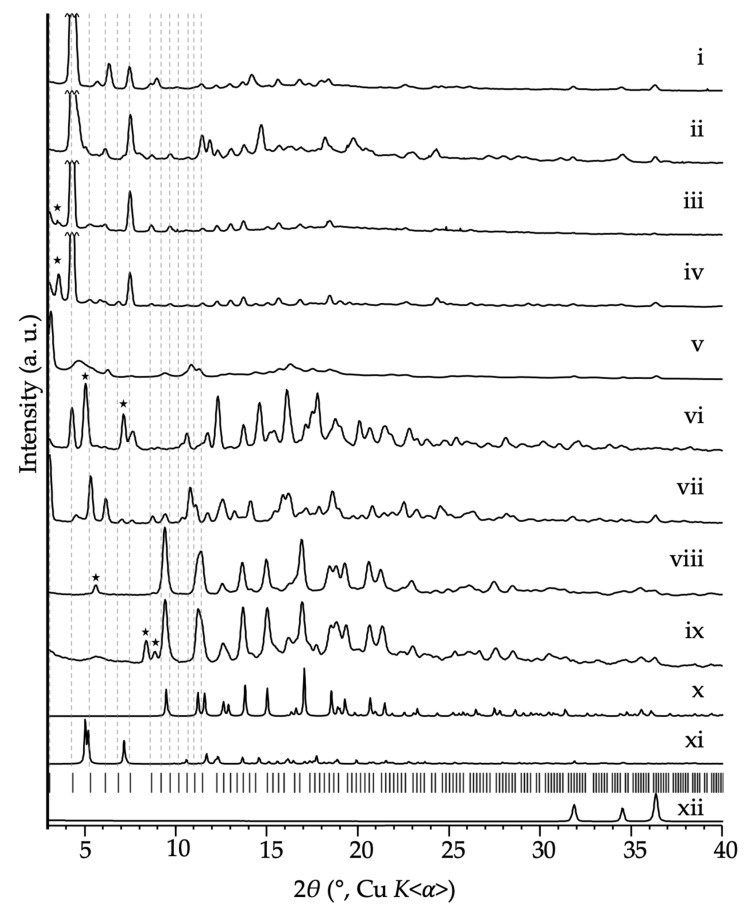
Experimental PXRD patterns of LAG-aging syntheses using nanoparticulate ZnO, imidazole, DEF, and selected cavitands, washed after *t* days. (i) cavitand = **MeHCH_2_**, *t* = 16 d, (ii) cavitand = **Me*i*BuCH_2_**, *t* = 16 d, (iii) cavitand = **BrPhCH_2_**, *t* = 7 d, (iv) cavitand = **BrC5CH_2_**, *t* = 5 d, (v) **HPhCH_2_**_,_ *t* = 12 d, (vi) cavitand = **MePhCH_2_** (*rctt*:*rccc* = 87:13), *t* = 5 d, (vii) cavitand = **MeHCH_2_-6**, *t* = 5 d, (viii) **MeHSiMe_2_**, *t* = 11 d, (ix) **H(4MePh)CH_2_**, *t* = 11 d, and (xii) nanoparticulate ZnO. Simulated PXRD patterns of (x) **cag**-Zn**Im**_2_ (VEJYUF) and (xi) **nog**-Zn**Im**_2_ (HIFWAV). Tick marks denote predicted peak positions of 0.90**MeMeCH_2_**@RHO-Zn_16_**Im**_32_. Stars denote small amounts of impurities that are not defined in Table 1.

A series of LAG-aging experiments using nanoparticulate zinc oxide, imidazole, and the putative template in the presence of DEF (molar ratio of 1:2:0.5:4) were performed. The reactions were monitored over time by PXRD and the products were worked up for analysis (chloroform washing) after near-complete consumption of ZnO. The PXRD patterns of the resulting final, chloroform-washed products are shown in Figure 5. The control reaction (see ref. [23]), using no added template, gave mainly **coi**-Zn**Im**_2_ (EQOCOC). It is worth noting that, for all mechanochemical ZIF syntheses, care must be taken to thoroughly clean the milling jars and equipment before every experiment. Due to the diverse topological landscape (framework polymorphism) of zinc imidazolate, it was found that residual seeds of Zn**Im**_2_ materials with various framework topologies can provide a competing templation effect, and sometimes results in the formation of mixtures. We are currently exploring intentional framework seeding as a possible means of controlling Zn**Im**_2_ framework polymorphism and/or achieving novel Zn**Im**_2_ framework polymorphs.

The framework topologies of the discernable ZIF products associated with each putative template are summarized in Table 1. In identifying the products of mechanochemical reactions, the region of the PXRD pattern below 10° 2*θ* (Cu *K*<α>) was particularly useful for establishing the presence of RHO-topology ZIFs, as there are up to nine observable (*hkl*) reflections that are characteristic of the RHO-Zn**Im**_2_ framework in this region. Many of the more dense Zn**Im**_2_ ZIF framework polymorphs exhibit much smaller units cells and therefore no (or few) low-angle peaks (e.g., **cag**-Zn**Im**_2_ (VEJYUF), **coi**-Zn**Im**_2_ (EQOCOC), **zni**-Zn**Im**_2_ (IMIDZB06), Zn_4_(H**Im**)**Im**_8_ (KUMXEW)). Other (non-RHO) low density framework polymorphs may exhibit low angle (*hkl*) reflections, but they are at discernably different 2*θ* positions from RHO-Zn**Im**_2_ (e.g*.,* **10mr**-Zn**Im**_2_ (GOQSIQ), MER-Zn**Im**_2_ (VEJZIU, EWENUR)). Peaks above ≈ 10–15° 2*θ* (Cu *K*<α>) in the PXRD pattern are less conclusive when it comes to identifying the topological products, as a number of ZIF framework polymorphs can give rise to (*hkl*) reflections at those 2*θ* angles, and RHO-Zn**Im**_2_ itself has many peaks in that region. It can therefore be difficult to establish whether the samples containing an RHO-Zn**Im**_2_ framework product also contain other more dense ZIF framework polymorphs as impurities. To assist in making that decision, an expanded comparison of each of the product PXRD patterns, alongside the predicted peaks positions for RHO-Zn**Im**_2_, is provided in the Supporting Information.

Another complication arises from the relative intensities of the PXRD peaks. It is important to recognize that the Zn**Im**_2_ framework topology can be assigned on the basis of the 2*θ* positions of the collective (*hkl*) reflections, where the relative peak intensities can vary widely depending upon how the electron density—associated with solvent (e.g., solvent identity and/or atomic scattering factors, position, order/disorder, or absence) or included cavitand template—is distributed within the pores of the ZIF framework. It has been reported, for instance, that the relative intensities of the two lowest angle peaks—the (100) and (110) peaks at 3.04° and 4.32° 2*θ*, respectively—of RHO-Zn**BIm**_2_ (ZIF-11) are dramatically affected by the occupancy of the pores by solvent [51]. Among other relative intensity differences, filling the Zn**BIm**_2_ (ZIF-11) pores (i.e., the LTA cages) with toluene was shown to dramatically increase the relative intensity of the (100) peak, while concomitantly lowering the relative intensity of the (110) peak.

The first modified template that was evaluated was **MeHCH_2_** (R = Me, R’ = H, Y = CH_2_). **MeHCH_2_** differs from the original **MeMeCH_2_** template only in the fact that it has a smaller lower rim substituent (H instead of CH_3_). It was hypothesized that the smaller H atoms should be less demanding with respect to the requisite conformational reorganization of the imidazolate ligands within the eight-rings of the template@*d8r* motifs (θ_3-4_, Figure 1). Thus, it was anticipated that **MeHCH_2_** would function similarly to **MeMeCH_2_** as a template for RHO-Zn**Im**_2_. Indeed, the LAG-aging procedure, after 16 days, resulted in near-quantitative conversion into the RHO topology product, isolated as 1.6**MeHCH_2_**@RHO-Zn_16_**Im**_32_ (Figure 5i). The relatively high degree of **MeHCH_2_** incorporation indicates that all accessible *d8r*s (half of the overall number are deemed accessible based on crystallographic analysis [23] of **MeMeCH_2_**@RHO-Zn_16_**Im**_32_) are occupied and that there is an excess of **MeHCH_2_** remaining in the structure, even after extensive washing. It seems likely that the excess **MeHCH_2_** is contained in the LTA cages, though it is also possible that the smaller size of **MeHCH_2_** compared to **MeMeCH_2_** may enable some of the *d8r*s that are deemed inaccessible to be filled with the smaller cavitand.

**Figure 6:**
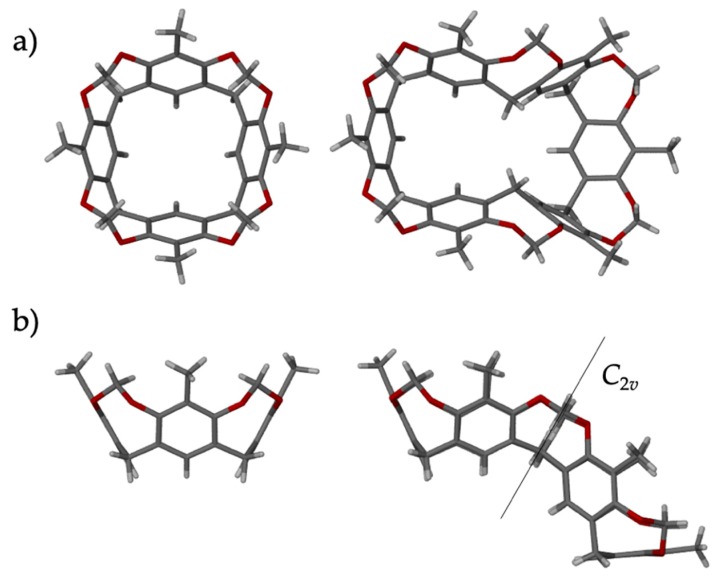
Comparison of the crystal structures of **MeHCH_2_** (left; CSD code VUWMIL [56]) and **MeHCH_2_-6** (right; CSD code QUCWOA [55], chloroform solvate, solvent molecules omitted for clarity), illustrating the similarities of a portion of their molecular structures. (**a**) top view, (**b**) side view.

Next, the **MeHCH_2_-6** cavitand (R = Me, R’ = H, Y = CH_2_, *n* = 6), containing six 2-methylresorcinol units, was used. The upper and lower rim substitution in **MeHCH_2_-6** is identical to that of **MeHCH_2_**, but the addition of two more resorcinol units makes the overall cavitand significantly larger and incompatible with the 4/8-fold symmetry of the *d8r*s. Clearly, also, **MeHCH_2_-6** cannot fit within a completely formed Zn**Im**_2_ *d8r* motif. However, **MeHCH_2_-6** exhibits a “double-bowl” type *C*_2*v*_ symmetric conformation in the solid state and in solution [55]. The conformation (Figure 6) is such that two narrow ends of the elongated, pinched double-bowl structure, mimic, almost perfectly, three of the four rings of the four-fold symmetric **MeHCH_2_-4** cavitand. Thus, it was of interest to determine whether **MeHCH_2_-6** can give rise to the RHO-Zn**Im**_2_ framework by templating only partial formation of *d8r* motif. PXRD analysis of the product of the LAG-aging synthesis using **MeHCH_2_-6** as a putative template (washed after 5 days) revealed that the main product was in fact an RHO-Zn**Im**_2_ framework ZIF, isolated as 5.8**MeHCH_2_-6**@RHO-Zn_16_**Im**_32_ (Figure 5vii). Notably, a significant portion of the **MeHCH_2_-6** template is retained of the MOF after washing. Yet the large **MeHCH_2_-6** macrocycle simply cannot fit within a *d8r* of the RHO-Zn**Im**_2_ structure. We conclude that the residual **MeHCH_2_-6** must be retained within the large LTA cage. This conclusion is also consistent with the relative intensities of the 5.8**MeHCH_2_-6**@RHO-Zn_16_**Im**_32_ PXRD pattern, which are quite different from the aforementioned 0.9**MeMeCH_2_**@RHO-Zn_16_**Im**_32_ and 1.6**MeHCH_2_**@RHO-Zn_16_**Im**_32_ (Figure 5i) materials. The most notable differences correspond to the two lowest angle peaks. The (100) peak is intense in 5.8**MeHCH_2_-6**@RHO-Zn_16_**Im**_32_ but it almost absent in 1.6**MeHCH_2_**@RHO-Zn_16_**Im**_32_. Conversely, the (110) peak is almost missing (and appears to be shifted to somewhat higher angle) in 5.8**MeHCH_2_-6**@RHO-Zn_16_**Im**_32_, but is the most intense peak in 1.6**MeHCH_2_**@RHO-Zn_16_**Im**_32_. These results suggest that only a portion of the requisite structural motif—in this case, the *d8r*—needs to be formed to have an influence on the topological outcome of the final framework. Thus, even though a template may not be able to fit entirely within a given desired motif (e.g., the *d8r*), it may still be capable of templating the desired structure by biasing the assembly process. Moreover, the ultimate location of the template in the final structure may not be a reflection of portion of the structure for which it exerts a structural influence.

Next, we explored **MeHSiMe_2_** (R = Me, R’ = H, Y = SiMe_2_). as a possible template. **MeHSiMe_2_** differs from the effective **MeHCH_2_** template in that the “Y” groups (Y = -SiMe_2_-) that connect the resorcinol units around the upper rim of the bowl are much larger, and make the rim of the bowl much wider. The dimethylsilyl moieties were expected to inhibit approach of the metal-coordinated imidazolate ligands to the eight ether oxygen atoms that function as hydrogen bond acceptors in the original template. Moreover, structural models suggest that the outwardly directed methylsilyl moieties cannot be sterically accommodated by the imidazolates of the eight-rings of the *d8r*s. Not surprisingly, **MeHSiMe_2_** proved not to be a functional template for the RHO-Zn**Im**_2_ framework. The LAG-aging synthesis using **MeHSiMe_2_** gave, after 11 days, a mixture of products, the majority component being **cag**-Zn**Im**_2_ (presumably originally occupied by DEF, Figure 5viii). We can conclude that broadening the top rim of the cavitand, and/or sterically preventing the ether oxygen atoms from hydrogen bonding with the imidazolate ligands, results in failure of the template.

Finally, we greatly broadened the lower rim of the cavitand bowl by using an *rctt* stereoisomer of **MePhCH_2_** (R = Me, R’ = Ph, Y = CH_2_). As mentioned, all other cavitand templates employed herein are in their stereopure *rccc*-forms (or have no stereoisomers: R’ = H), where all R’ groups point “down” from the bowls (axial with respect to the macrocylic ring), parallel to the *C*_4_ axis. In the *rctt* form of **MePhCH_2_**, however, two adjacent phenyl rings are directed approximately orthogonal (equatorial to the macrocylic ring) to the cavitand bowl. As two of the bottom rim substituents are protruding horizontally outward, the effective diameter of the lower rim substituents of any *rctt* cavitand is much larger than its *rccc* stereoisomer. Clearly, like **MeHCH_2_-6**, *rctt*-**MePhCH_2_** cannot fit within a fully formed d8r Zn**Im**_2_ motif, but assembly of up to half of the d8r may be possible. When **MePhCH_2_** cavitand, enriched in the *rctt* stereoisomer (*rctt*:*rccc* = 87:13), was used as a template in a LAG-aging synthesis, the washed product after 5 days proved to be a mixture of **nog**-Zn**Im**_2_ and *x***MePhCH_2_**@RHO-Zn_16_**Im**_32_ (Figure 5vi). Remarkably, the product was found by ^1^H NMR spectroscopy to have incorporated both the *rccc* and the *rctt* forms of **MePhCH_2_**, in approximately the same ratio as the starting cavitand mixture (Figure S24). As **nog**-Zn**Im**_2_ is not sufficiently porous to accommodate either cavitand, we conclude they are embedded within the RHO-Zn**Im**_2_ portion of the product. We hypothesize that, similar to the templation of RHO-Zn**BIm**_2_ by **MeMeCH_2_**, and RHO-Zn**Im**_2_ by **MeHCH_2_-6**, both the *rctt* cavitand and the *rccc* cavitand can initiate templation of the RHO framework through at least partial formation of *d8r* motifs. Though it may be possible for *rccc*-**MePhCH_2_** to be encapsulated by the *d8r*s, *rctt*-**MePhCH_2_** clearly cannot be, and therefore must reside within the LTA cages. Clearly, though, and not surprisingly, *rctt*-**MePhCH_2_** is not a very effective template for formation of the RHO-Zn**Im**_2_ framework, as a similar amount of **nog**-Zn**Im**_2_ is formed in this reaction.

It appeared that changes to the upper rim of the cavitand affected the templation of *d8r*s and RHO-Zn**Im**_2_ more than changes to the bottom rim. Since templation is based on hydrogen bonding interactions, and the only hydrogen bond donors in the cavitand molecule are the oxygen atoms nearer the upper rim, this observation is not particularly surprising. The question then remained: keeping the basic backbone of the cavitand the same (four-membered macrocyclic ring, *rccc* stereochemistry, Y = CH_2_ bridging substituents), are there limitations on the size of substituents on the upper, R, and lower, R’, rims of the cavitand?

To test this, we first increased the bulk of the lower rim substituents, exploring **Me*i*BuCH_2_** (R = Me, R’ = *i*-Bu, Y = CH_2_) as a template. The LAG-aging synthesis resulted in mainly 0.3**Me*i*BuCH_2_**@RHO-Zn_16_**Im**_32_ after 16 days of aging, as confirmed by PXRD and NMR analysis (Figure 5ii). Similar to 0.2**MeMeCH_2_**@RHO-Zn_16_(**Cl_2_Im**)_32_, only one tenth of the *d8*rs (or one fifth of the ‘accessible’ *d8r* motifs) are occupied by cavitand. This is likely due to the steric bulk of **MeiBuCH_2_**, limiting the amount of cavitand that is ultimately incorporated.

If a phenyl-footed cavitand, namely **HPhCH_2_** (R = H, R’ = Ph, Y = CH_2_), is used, the LAG-aging experiment results in 0.8**HPhCH_2_**@Zn_16_**Im**_32_ after 12 days of aging, as confirmed by NMR analysis. The PXRD pattern of this material PXRD is unusual (Figure 5v) in that the peaks are broad, but the peak positions are nonetheless almost entirely consistent with the RHO-Zn**Im**_2_ framework topology. The relative intensities of the peaks are unusual as compared to the other RHO-Zn**Im**_2_ materials. Namely, the (100) peak (at 3.06° 2*θ*), which is very small in PXRD patterns of 1.6**MeHCH_2_**@RHO-Zn_16_**Im**_32_ and 0.9**MeMeCH_2_**@RHO-Zn_16_**Im**_32_, is very intense, whereas the (110) peak, which is the dominant peak in 1.6**MeHCH_2_**@RHO-Zn_16_**Im**_32_ and 0.9**MeMeCH_2_**@RHO-Zn_16_**Im**_32_, is much broader and of lower maximum intensity. The pattern more resembles that of 5.8**MeHCH_2_-6**@RHO-Zn_16_**Im**_32_, but is more broad. We hypothesize that these effects could be due to the large quantity of the relatively bulky **HPhCH_2_** cavitand trapped inside the *d8r*s of the material. The level of incorporation of the **HPhCH_2_** is four times that of **Me*i*BuCH_2_**. Though this might seem counterintuitive due to the fact that a phenyl group contains 6 carbon atoms, and an isobutyl group only 4, the effective total “width” of an isobutyl group is surely larger than that of a phenyl group due to the conformational degrees of freedom. Whereas the isobutyl group is branched at the C_2_ position, however, the phenyl group is branched at C_1_. Thus, the local steric ramifications (near the eight-rings of the framework) associated with accommodating the bulky tetraphenyl-substituted end of the cavitand within the *d8r*s could cause local twisting and/or ‘breathing’-like structural perturbations of the affected 8-rings of the framework. Such local structural perturbations may be responsible for broadening of the peaks. Lastly, the relatively high intensity of the (100) peak may be due to protrusion of the phenyl feet into the LTA cage.

Finally, we explored **H(4MePh)CH_2_** (R = H, R’ = 4-MePh, Y = CH_2_) as a putative template. Compared to **HPhCH_2_**, **H(4MePh)CH_2_** is simply rigidly extended at the phenyl feet (R’) by one methyl group. Remarkably, after 11 days, the LAG-aging reaction products proved to be a mixture of zinc imidazolates, but mainly **cag**-Zn**Im_2_** (presumably originally occupied by DEF, Figure 5ix), containing no traces of an RHO-Zn**Im_2_** framework product. We therefore conclude that cavitands with 4-methylphenyl (or longer) feet are too long to successfully template RHO-Zn**Im_2_**. The result is somewhat surprising considering that **H(4MePh)CH_2_** possesses the same core molecular architecture as **HPhCH_2_**, which appears to template formation of an RHO-Zn**Im_2_** product, and the observation that the ability to be encapsulated by the framework does not seem not to be a prerequisite for a successful cavitand RHO template.

Having explored the effects of changing the size of substituents on the lower rim ‘feet’ of the cavitand, we turned toward increasing the size of the upper rim substituents. We employed two different cavitands in the LAG-aging procedure, namely **BrPhCH_2_** (R = Br, R’ = Ph, Y = CH_2_) and **BrC5CH_2_** (R = Br, R’ = *n*-C_5_H_11_, Y = CH_2_), each possessing bromine atoms at the upper rim. Structural models suggest that the upper rim bromine atoms may interact favorably with the imidazolate ligands of the *d8r* eight-rings. At the lower rim are phenyl groups or *n*-pentyl substituents. **BrPhCH_2_** has analogous feet to the successful **HPhCH_2_** template, and **BrC5CH_2_** possesses feet with the same approximate total length of the unsuccessful **H(4MePh)CH_2_** template (five-carbon atoms), but its feet are flexible. Based on PXRD (Figure 5iii,iv) and ^1^H-NMR analysis, both cavitands successfully yielded high quality RHO-Zn**Im**_2_ framework products, as 0.2**BrC5CH_2_**@RHO-Zn_16_**Im**_32_ (after 5 days), and 0.6**BrPhCH_2_**@RHO-Zn_16_**Im**_32_ (after 7 days), respectively. Higher incorporation of the phenyl-footed cavitand, compared to the pentyl cavitand, follows the trend set by **Me*i*BuCH_2_** and **HPhCH_2_**, where the flat arene feet seem to allow for greater cavitand incorporation than the nominally smaller but conformationally flexible, and thus potentially bulkier, aliphatic feet.

## 3. Materials and Methods

Full details of all experimental procedures and characterizations can be found as Supplementary Information. In a typical LAG-aging experiment, nanoparticulate zinc oxide, imidazole ligand, the cavitand template, and DEF were mixed in a 1:2:0.5:4 molar ratio, and milled in a poly(methyl methacrylate) jar (Form-Tech Scientific, Montreal, QC, Canada) for 2 min at 30 Hz. The **MeMeCH_2_** [32] cavitand was used to template reactions employing 4,5-dichloroimidazole (H**Cl_2_Im**), benzimidazole (H**BIm**), 5-nitrobenzimidazole (H**NO_2_BIm**), 5,6-dimethylbenzimidazole (H**Me_2_BIm**), and theophylline (**Theo**). Nine cavitands were explored as possible templates in reaction with the simple imidazole (H**Im**) ligand: **HPhCH_2_** [57], **H(4-MePh)CH_2_** [57], **MeHCH_2_** [55], **MeHCH_2_-6** [55], **MeHSiMe_2_** [58], **Me*i*BuCH_2_** [56], **MePhCH_2_** [59], **BrC5CH_2_** [60] and **BrPhCH_2_** [57]. All cavitands were prepared according to literature procedures [32] and were used in unsolvated form, after drying in a vacuum oven (Precision Scientific, Chicago, IL, USA) at 120 °C for 24 h. The resulting dense paste was transferred to a 5 mL glass vial and left to age. Reaction progress was monitored by PXRD. When the reaction had reached completion, as evidenced by the disappearance of ZnO peaks from the PXRD pattern, the sample was washed extensively with chloroform according to a procedure previously shown to remove all excess template, and the product was analyzed by PXRD and NMR to confirm product topology and determine the cavitand incorporation ratio, respectively. Control experiments were performed for syntheses using H**Cl_2_Im** and H**BIm** by conducting the LAG-aging procedure without the addition of the **MeMeCH_2_** template. Low temperature and pressure (T = 77 K, *p* < 1 bar) nitrogen adsorption measurements for the synthesized RHO topology zinc 4,5-dichloroimidazolate and zinc benzimidazolate were performed using a Quantachrome Autosorb 1 (Quantachrome Instruments, Boynton Beach, Florida, USA; now Anton Paar QuantaTec Inc.) volumetric analyzer.

## 4. Conclusions

In conclusion, it has been shown that **MeMeCH_2_**, a macrocylic cavitand that acts as a ‘shoe-last’ template for framework *d8r*s in the mechanochemical synthesis of *x***MeMeCH_2_**@RHO-Zn_16_**Im**_32_ (the first example of an RHO-Zn**Im**_2_ framework ZIF), can be used to accelerate the synthesis of other RHO-ZnR**Im**_2_ frameworks. Employing a LAG-aging templation approach with **MeMeCH_2_** allowed preparation of 0.2**MeMeCH_2_**@RHO-Zn_16_(**Cl_2_Im**)_32_ (*x***MeMeCH_2_**@ZIF-71) and RHO-Zn**BIm**_2_ (ZIF-11), the latter framework being prepared by mechanochemical methods for the first time. The mechanochemically prepared sample of RHO-Zn**BIm**_2_ (ZIF-11), which had long been thought to be nonporous to N_2_ at 77 K, appears to be open to dinitrogen sorption under these conditions, revealing it to have a high surface area. It has further been shown that several other related macrocyclic cavitands—**MeHCH_2_**, **MeHCH_2_-6**, **HPhCH_2_**, **Me*i*BuCH_2_**, **BrC5CH_2_**, and **BrPhCH_2_**—can serve as templates for the mechanochemical synthesis of *x*(cavitand)@RHO-Zn**Im**_2_ ZIFs, each being encapsulated to varying degrees by the framework. Notably, even cavitands that cannot be fully encapsulated by the *d8r* motifs in the final products can serve as templates for at least partial assembly of these motifs and the overall RHO framework topology, and must thereby reside in defect sites or, more likely, in the LTA cages of the framework. Footed cavitands with *rctt* stereochemistry, not surprisingly, act as poor templates for the RHO-Zn**Im**_2_ framework, but may still be encapsulated. Cavitands with bulky—e.g., dimethylsilyl—moieties bridging the resorcinol units appear to inhibit the C-H···O hydrogen bonding interaction responsible for the templation effect, preventing successful formation the RHO-Zn**Im**_2_ framework. Similarly, bottom rim substituents should be smaller/shorter than rigid 4-methylphenyl groups. The wide variety of cavitands that can be used for templation of RHO topology zinc imidazolates with no change in synthetic protocol indicates that cavitand functionalization might be a good way to introduce desired elements, such as useful functional groups or catalytically active moieties, into the pores of high porosity RHO-framework ZIFs. More broadly, the results suggest that a wide array of small molecules may be chosen or designed to direct the topological outcome in the synthesis of ZIFs susceptible to framework polymorphism. Besides representing a step forward towards topologically-controlled synthesis of zeolitic metal-organic frameworks, the presented work is also a notable contribution to mechanochemical and mechanically-activated synthesis of complex structures, as it represents a still rare example of using templates to direct the outcome of solvent-less synthetic procedures [61].

## Figures and Tables

**Table 1:** Summary of cavitand putative templates explored in the LAG-aging reactions between zinc oxide, imidazole, DEF, and **RR’Y**-*n* cavitand (Figure 1), as well as the framework topological products of the reactions.

R	R’	Y	*n*	Stereochemistry	Topological Result
**H**	**Ph**	**CH_2_**	4	*rccc*	RHO
**H**	**4-MePh**	**CH_2_**	4	*rccc*	**cag**
**Me**	**H**	**CH_2_**	4	*rccc*	RHO
**Me**	**H**	**CH_2_**	6	*rccc*	RHO
**Me**	**H**	**SiMe_2_**	4	*rccc*	**cag**
**Me**	***i*-Bu**	**CH_2_**	4	*rccc*	RHO
**Me**	**Ph**	**CH_2_**	4	87% *rctt*: 13% *rccc*	**nog** + RHO
**Br**	***n*-C_5_H_11_**	CH_2_	4	*rccc*	RHO
**Br**	**Ph**	**CH_2_**	4	*rccc*	RHO

## Supplementary Materials

Full details of all experimental procedures, assigned ^1^H-NMR spectra, detailed PXRD patterns for all prepared materials, and sorption analysis details for 0.2**MeMeCH_2_**@RHO-Zn_16_(**Cl_2_Im**)_32_ and RHO-Zn**BIm_2_** are available online.

## References

[1] Tian Y., Cai C., Ji Y., You X., Peng S., Lee G. (2002). [Co_5_(Im)_10_⋅2MB]∞: A Metal-Organic Open-Framework with Zeolite-Like Topology. Angew. Chem. Int. Ed..

[2] Park K.S., Ni Z., Côté A.P., Choi J.Y., Huang R., Uribe-Romo F.J., Chae H.K., O’Keeffe M., Yaghi O.M. (2006). Exceptional Chemical and Thermal Stability of Zeolitic Imidazolate Frameworks. Proc. Natl. Acad. Sci. USA.

[3] Eddaoudi M., Sava D.F., Eubank J.F., Adil K., Guillerm V. (2015). Zeolite-like Metal-Organic Frameworks (ZMOFs): Design, Synthesis, and Properties. Chem. Soc. Rev..

[4] Tan Y.X., Wang F., Zhang J. (2018). Design and Synthesis of Multifunctional Metal-Organic Zeolites. Chem. Soc. Rev..

[5] Wu T., Bu X., Zhang J., Feng P. (2008). New Zeolitic Imidazolate Frameworks: From Unprecedented Assembly of Cubic Clusters to Ordered Cooperative Organization of Complementary Ligands. Chem. Mater..

[6] Zhang J., Wu T., Zhou C., Chen S., Feng P., Bu X. (2009). Zeolitic Boron Imidazolate Frameworks. Angew. Chem. Int. Ed..

[7] O’Keeffe M., Peskov M.A., Ramsden S.J., Yaghi O.M. (2008). The Reticular Chemistry Structure Resource (RCSR) Database of, and Symbols for, Crystal Nets. Acc. Chem. Res..

[8] Moosavi S.M., Boyd P.G., Sarkisov L., Smit B. (2018). Improving the Mechanical Stability of Metal-Organic Frameworks Using Chemical Caryatids. ACS Cent. Sci..

[9] Noh K., Lee J., Kim J. (2018). Compositions and Structures of Zeolitic Imidazolate Frameworks. Isr. J. Chem..

[10] Ramirez J.R., Yang H., Kane C.M., Ley A.N., Holman K.T. (2016). Reproducible Synthesis and High Porosity of Mer-Zn(Im)_2_ (ZIF-10): Exploitation of an Apparent Double-Eight Ring Template. J. Am. Chem. Soc..

[11] Friščić T., Halasz I., Beldon P.J., Belenguer A.M., Adams F., Kimber S.A.J., Honkimäki V., Dinnebier R.E. (2013). Real-Time and in Situ Monitoring of Mechanochemical Milling Reactions. Nat. Chem..

[12] Akimbekov Z., Katsenis A.D., Nagabhushana G.P., Ayoub G., Arhangelskis M., Morris A.J., Friščić T., Navrotsky A. (2017). Experimental and Theoretical Evaluation of the Stability of True MOF Polymorphs Explains Their Mechanochemical Interconversions. J. Am. Chem. Soc..

[13] Tian Y.Q., Yao S.Y., Gu D., Cui K.H., Guo D.W., Zhang G., Chen Z.X., Zhao D.Y. (2010). Cadmium Imidazolate Frameworks with Polymorphism, High Thermal Stability, and a Large Surface Area. Chem. Eur. J..

[14] Morris W., Leung B., Furukawa H., Yaghi O.K., He N., Hayashi H., Houndonougbo Y., Asta M., Laird B.B., Yaghi O.M. (2010). A Combined Experimental-Computational Investigation of Carbon Dioxide Capture in a Series of Isoreticular Zeolitic Imidazolate Frameworks. J. Am. Chem. Soc..

[15] Banerjee R., Furukawa H., Britt D., Knobler C., O’Keeffe M., Yaghi O.M. (2009). Control of Pore Size and Functionality in Isoreticular Zeolitic Imidazolate Frameworks and Their Carbon Dioxide Selective Capture Properties. J. Am. Chem. Soc..

[16] Tian Y.-Q.Q., Cai C.-X.X., Ren X.-M.M., Duan C.-Y.Y., Xu Y., Gao S., You X.-Z.Z. (2003). The Silica-Like Extended Polymorphism of Cobalt(II) Imidazolate Three-Dimensional Frameworks: X-Ray Single-Crystal Structures and Magnetic Properties. Chem. Eur. J..

[17] Tian Y.Q., Zhao Y.M., Chen Z.X., Zhang G.N., Weng L.H., Zhao D.Y. (2007). Design and Generation of Extended Zeolitic Metal-Organic Frameworks (ZMOFs): Synthesis and Crystal Structures of Zinc(II) Imidazolate Polymers with Zeolitic Topologies. Chem. Eur. J..

[18] Shi Q., Kang X., Shi F.N., Dong J. (2015). Zn_10_(Im)_20_·4DBF: An Unprecedented 10-Nodal Zeolitic Topology with a 10-MR Channel and 10 Crystallographically Independent Zn Atoms. Chem. Commun..

[19] Shi Q., Xu W.J., Huang R.K., Zhang W.X., Li Y., Wang P., Shi F.N., Li L., Li J., Dong J. (2016). Zeolite CAN and AFI-Type Zeolitic Imidazolate Frameworks with Large 12-Membered Ring Pore Openings Synthesized Using Bulky Amides as Structure-Directing Agents. J. Am. Chem. Soc..

[20] Lalonde M.B., Mondloch J.E., Deria P., Sarjeant A.A., Al-Juaid S.S., Osman O.I., Farha O.K., Hupp J.T. (2015). Selective Solvent-Assisted Linker Exchange (SALE) in a Series of Zeolitic Imidazolate Frameworks. Inorg. Chem..

[21] Fei H., Cahill J.F., Prather K.A., Cohen S.M. (2013). Tandem Postsynthetic Metal Ion and Ligand Exchange in Zeolitic Imidazolate Frameworks. Inorg. Chem..

[22] Yang J., Zhang Y.-B.B., Liu Q., Trickett C.A., Gutiérrez-Puebla E., Monge M.Á., Cong H., Aldossary A., Deng H., Yaghi O.M. (2017). Principles of Designing Extra-Large Pore Openings and Cages in Zeolitic Imidazolate Frameworks. J. Am. Chem. Soc..

[23] Brekalo I., Kane C.M., Ley A.N., Ramirez J.R., Friščić T., Holman K.T. (2018). Use of a “Shoe-Last” Solid-State Template in the Mechanochemical Synthesis of High-Porosity RHO-Zinc Imidazolate. J. Am. Chem. Soc..

[24] Bennett T.D., Horike S. (2018). Liquid, Glass and Amorphous Solid States of Coordination Polymers and Metal–Organic Frameworks. Nat. Rev. Mater..

[25] Widmer R.N., Lampronti G.I., Chibani S., Wilson C.W., Anzellini S., Farsang S., Kleppe A.K., Casati N.P.M., Macleod S.G., Redfern S.A.T. (2019). Rich Polymorphism of a Metal-Organic Framework in Pressure-Temperature Space. J. Am. Chem. Soc..

[26] Guo X., Geng S., Zhuo M., Chen Y., Zaworotko M.J., Cheng P., Zhang Z. (2019). The Utility of the Template Effect in Metal-Organic Frameworks. Coord. Chem. Rev..

[27] Dong K., Zhang S., Wang J. (2016). Understanding the Hydrogen Bonds in Ionic Liquids and Their Roles in Properties and Reactions. Chem. Commun..

[28] Higgins E.M., Sherwood J.A., Lindsay A.G., Armstrong J., Massey R.S., Alder R.W., O’Donoghue A.C. (2011). PKas of the Conjugate Acids of N-Heterocyclic Carbenes in Water. Chem. Commun..

[29] Karagiaridi O., Lalonde M.B., Bury W., Sarjeant A.A., Farha O.K., Hupp J.T. (2012). Opening ZIF-8: A Catalytically Active Zeolitic Imidazolate Framework of Sodalite Topology with Unsubstituted Linkers. J. Am. Chem. Soc..

[30] Morabito J.V., Chou L.Y., Li Z., Manna C.M., Petroff C.A., Kyada R.J., Palomba J.M., Byers J.A., Tsung C.K. (2014). Molecular Encapsulation beyond the Aperture Size Limit through Dissociative Linker Exchange in Metal-Organic Framework Crystals. J. Am. Chem. Soc..

[31] Ye J.W., Zhou H.L., Liu S.Y., Cheng X.N., Lin R.B., Qi X.L., Zhang J.P., Chen X.M. (2015). Encapsulating Pyrene in a Metal-Organic Zeolite for Optical Sensing of Molecular Oxygen. Chem. Mater..

[32] Cram D.J., Karbach S., Kim H.E., Knobler C.B., Maverick E.F., Ericson J.L., Helgeson R.C. (1988). Host-Guest Complexation. 46. Cavitands as Open Molecular Vessels Form Solvates. J. Am. Chem. Soc..

[33] Mottillo C., Friščić T. (2017). Advances in Solid-State Transformations of Coordination Bonds: From the Ball Mill to the Aging Chamber. Molecules.

[34] Friščić T., Halasz I., Štrukil V., Eckert-Maksić M., Dinnebier R.E. (2012). Clean and Efficient Synthesis Using Mechanochemistry: Coordination Polymers, Metal-Organic Frameworks and Metallodrugs. Croat. Chem. Acta.

[35] Chen D., Zhao J., Zhang P., Dai S. (2019). Mechanochemical Synthesis of Metal–Organic Frameworks. Polyhedron.

[36] Užarević K., Wang T.C., Moon S.Y., Fidelli A.M., Hupp J.T., Farha O.K., Friščić T. (2016). Mechanochemical and Solvent-Free Assembly of Zirconium-Based Metal-Organic Frameworks. Chem. Commun..

[37] Julien P.A., Užarević K., Katsenis A.D., Kimber S.A.J., Wang T., Farha O., Zhang Y., Casaban J., Germann L.S., Etter M. (2016). In Situ Monitoring and Mechanism of the Mechanochemical Formation of a Microporous MOF-74 Framework. J. Am. Chem. Soc..

[38] James S.L., Adams C.J., Bolm C., Braga D., Collier P., Frišcic T., Grepioni F., Harris K.D.M., Hyett G., Jones W. (2012). Mechanochemistry: Opportunities for New and Cleaner Synthesis. Chem. Soc. Rev..

[39] Beldon P.J., Fábián L., Stein R.S., Thirumurugan A., Cheetham A.K., Friščić T. (2010). Rapid Room-Temperature Synthesis of Zeolitic Imidazolate Frameworks Using Mechanochemistry. Angew. Chem. Int. Ed..

[40] Katsenis A.D., Puškarić A., Štrukil V., Mottillo C., Julien P.A., Užarević K., Pham M.-H.H., Do T.-O.O., Kimber S.A.J.J., Lazić P. (2015). In Situ X-Ray Diffraction Monitoring of a Mechanochemical Reaction Reveals a Unique Topology Metal-Organic Framework. Nat. Commun..

[41] Mottillo C., Lu Y., Pham M.H., Cliffe M.J., Do T.O., Friščić T. (2013). Mineral Neogenesis as an Inspiration for Mild, Solvent-Free Synthesis of Bulk Microporous Metal-Organic Frameworks from Metal (Zn, Co) Oxides. Green Chem..

[42] Cliffe M.J., Mottillo C., Stein R.S., Bučar D.K., Friščić T. (2012). Accelerated Aging: A Low Energy, Solvent-Free Alternative to Solvothermal and Mechanochemical Synthesis of Metal-Organic Materials. Chem. Sci..

[43] Tanaka S., Kida K., Nagaoka T., Ota T., Miyake Y. (2013). Mechanochemical Dry Conversion of Zinc Oxide to Zeolitic Imidazolate Framework. Chem. Commun..

[44] Brekalo I., Yuan W., Mottillo C., Lu Y., Zhang Y., Casaban J., Holman K.T., James S.L., Duarte F., Williams P.A. (2020). Manometric Real-Time Studies of the Mechanochemical Synthesis of Zeolitic Imidazolate Frameworks. Chem. Sci..

[45] Khay I., Chaplais G., Nouali H., Ortiz G., Marichal C., Patarin J. (2016). Assessment of the Energetic Performances of Various ZIFs with SOD or RHO Topology Using High Pressure Water Intrusion-Extrusion Experiments. Dalt. Trans..

[46] Houndonougbo Y., Signer C., He N., Morris W., Furukawa H., Ray K.G., Olmsted D.L., Asta M., Laird B.B., Yaghi O.M. (2013). A Combined Experimental–Computational Investigation of Methane Adsorption and Selectivity in a Series of Isoreticular Zeolitic Imidazolate Frameworks. J. Phys. Chem. C.

[47] Forman E.M., Baniani A., Fan L., Ziegler K.J., Zhou E., Zhang F., Lively R.P., Vasenkov S. (2020). Relationship between Ethane and Ethylene Diffusion inside ZIF-11 Crystals Confined in Polymers to Form Mixed-Matrix Membranes. J. Memb. Sci..

[48] Taylor M.K., Runčevski T., Oktawiec J., Gonzalez M.I., Siegelman R.L., Mason J.A., Ye J., Brown C.M., Long J.R. (2016). Tuning the Adsorption-Induced Phase Change in the Flexible Metal-Organic Framework Co(Bdp). J. Am. Chem. Soc..

[49] Yang Q.-F., Cui X.-B., Yu J.-H., Lu J., Yu X.-Y., Zhang X., Xu J.-Q., Hou Q., Wang T.-G. (2008). A Series of Metal–Organic Complexes Constructed from in Situ Generated Organic Amines. CrystEngComm.

[50] Jayaramulu K., Masa J., Morales D.M., Tomanec O., Ranc V., Petr M., Wilde P., Chen Y.T., Zboril R., Schuhmann W. (2018). Ultrathin 2D Cobalt Zeolite-Imidazole Framework Nanosheets for Electrocatalytic Oxygen Evolution. Adv. Sci..

[51] He M., Yao J., Liu Q., Zhong Z., Wang H. (2013). Toluene-Assisted Synthesis of RHO-Type Zeolitic Imidazolate Frameworks: Synthesis and Formation Mechanism of ZIF-11 and ZIF-12. Dalt. Trans..

[52] Noguera-Díaz A., Villarroel-Rocha J., Ting V.P., Bimbo N., Sapag K., Mays T.J. (2019). Flexible ZIFs: Probing Guest-Induced Flexibility with CO_2_, N_2_ and Ar Adsorption. J. Chem. Technol. Biotechnol..

[53] Germann L.S., Katsenis A.D., Huskić I., Julien P.A., Užarević K., Etter M., Farha O.K., Friščić T., Dinnebier R.E. (2020). Real-Time in Situ Monitoring of Particle and Structure Evolution in the Mechanochemical Synthesis of UiO-66 Metal–Organic Frameworks. Cryst. Growth Des..

[54] Wu T., Bu X., Liu R., Lin Z., Zhang J., Feng P. (2008). A New Zeolitic Topology with Sixteen-Membered Ring and Multidimensional Large Pore Channels. Chem. Eur. J..

[55] Naumann C., Román E., Peinador C., Ren T., Patrick B.O., Kaifer A.E., Sherman J.C. (2001). Expanding Cavitand Chemistry: The Preparation and Characterization of [n]Cavitands with n ≥ 4. Chem. Eur. J..

[56] Kane C.M., Ugono O., Barbour L.J., Holman K.T. (2015). Many Simple Molecular Cavitands Are Intrinsically Porous (Zero-Dimensional Pore) Materials. Chem. Mater..

[57] Cram D.J., Stewart K.D., Goldberg I., Trueblood K.N. (1989). Host-Guest Complexation. 49. Cavitands Containing Two Binding Cavities. J. Am. Chem. Soc..

[58] Kane C.M., Banisafar A., Dougherty T.P., Barbour L.J., Holman K.T. (2016). Enclathration and Confinement of Small Gases by the Intrinsically 0D Porous Molecular Solid, Me,H,SiMe_2_. J. Am. Chem. Soc..

[59] Kane C.M. (2015). Crystalline Organic Cavitands As Microcavity Materials. Ph.D. Thesis.

[60] Bryant J.A., Blanda M.T., Vincenti M., Cram D.J., Donegani G., Fauser V.G. (1991). Guest Capture during Shell Closure. J. Am. Chem. Soc..

[61] Kaabel S., Stein R.S., Fomitšenko M., Järving I., Friščić T., Aav R. (2019). Size-Control by Anion Templating in Mechanochemical Synthesis of Hemicucurbiturils in the Solid State. Angew. Chem. Int. Ed..

