# Supplementary material for: Exploring the Scope of Macrocyclic “Shoe-last” Templates in the Mechanochemical Synthesis of RHO Topology Zeolitic Imidazolate Frameworks (ZIFs)
Source: Molecules. 2020 Feb 1;25(3):633. doi: 10.3390/molecules25030633 (PMC7037713; doi:10.3390/molecules25030633)
Supplement: Supplementary file 1 [file molecules-25-00633-s001.pdf]

Supporting Information for

Exploring the scope of macrocyclic "shoe-last" templates in the mechanochemical synthesis of RHO topology Zeolitic Imidazolate Frameworks (ZIFs)

Ivana Brekalo,[1] David Deliz[1], Christopher M. Kane[1], Tomislav Friščić*,[2], K. Travis Holman*,[1]

[1]Department of Chemistry, Georgetown University, Washington, D.C. 20057, United States

[2]Department of Chemistry, McGill University, Montreal. QC, H3A 0B8, Canada

Abstract: The macrocyclic cavitand **MeMeCH₂** is used as a template for the mechanochemical synthesis of 0.2**MeMeCH₂**@RHO-Zn_{16}(**Cl_2Im**)$_{32}$ (0.2**MeMeCH₂**@ZIF-71) and RHO-Zn**BIm**$_2$ (ZIF-11) ZIFs. It is shown that **MeMeCH₂** significantly accelerates the mechanochemical synthesis, providing high porosity products (BET surface areas of 1140 m^2/g and 869 m^2/g, respectively). Templation of RHO-topology ZIF frameworks constructed of linkers larger than benzimidazole (H**BIm**) was unsuccessful. It is also shown that cavitands other than **MeMeCH₂** —namely **MeHCH₂**, **Me*i*BuCH₂**, **HPhCH₂**, **MePhCH₂**, **BrPhCH₂**, **BrC5CH₂**—can serve as effective templates for the synthesis of *x*(cavitand)@RHO-Zn**Im**$_2$ products. The limitations on cavitand size and shape are explored.

TABLE OF CONTENTS

1. EXPERIMENTAL DETAILS 2

1.1 General Details 2

1.2 Instrumental Details 2

2. SYNTHESIS AND CHARACTERIZATION OF RHO TOPOLOGY SUBSTITUTED ZINC IMIDAZOLATES USING **$MeMeCH_2$** AS A TEMPLATE 4

2.1. Synthesis of 0.2**$MeMeCH_2$**@RHO-Zn_{16}(**Cl_2Im**)$_{32}$ 4

2.2. Control reaction and time monitoring of the LAG Procedure synthesis of 0.2**$MeMeCH_2$**@RHO-Zn_{16}(**Cl_2Im**)$_{32}$ 6

2.3. Mechanochemical synthesis of RHO-Zn**BIm_2** (ZIF-11) 8

2.4. Control reaction and time monitoring of the LAG Procedure synthesis of RHO-Zn**BIm_2** 10

2.5. Templated mechanochemical experiments using **$MeMeCH_2$** and other ligands 12

3. SYNTHESIS AND CHARACTERIZATION OF RHO TOPOLOGY ZINC IMIDAZOLATES USING OTHER CAVITAND TEMPLATES 13

3.1. LAG-aging reaction using **$HPhCH_2$** as template 13

3.2. LAG-aging reaction using **H(4MePh)CH_2** as template 15

3.3. LAG-aging reaction using **$MeHCH_2$** as template 15

3.4. LAG-aging reaction using **$MeHCH_2$-6** as template 17

3.5. LAG-aging reaction using **$MeHSiMe_2$** as template 18

3.6. LAG-aging reaction using **Me*i*BuCH_2** as template 19

3.7. LAG-aging reaction using **$MePhCH_2$** as template 21

3.8. LAG-aging reaction using **$BrC5CH_2$** as template 22

3.9. LAG-aging reaction using **$BrPhCH_2$** as template 24

4. REFERENCES 25

1. EXPERIMENTAL DETAILS

1.1 General Details

Nanoparticulate zinc oxide (nano-ZnO, from NanoTek, 40-100 nm), imidazole (99%), 4,5-dichloroimidazole (98%), benzimidazole (98%), 5-nitrobenzimidazole (98%), 5,6-dimethylbenzimidazole (98%), and theophylline monohydrate (99%) were purchased from Alfa Aesar. N,N-diethylformamide (DEF, 99%), and deuterium chloride (35 wt. % solution in D_2O, 99 atom % D) were purchased from Sigma-Aldrich. Chloroform (ACS cert.) was purchased from Fisher Scientific. Deuterated chloroform (D, 99.8%) and dimethylsulfoxide (D, 99.9%) were purchased from Cambridge Isotope Laboratories. All chemicals were used without further purification.

Anhydrous theophylline was prepared by heating theophylline monohydrate in a vacuum oven at 125 ℃. The cavitands used - **HPhCH$_2$**,[1] **H(4MePh)CH$_2$**,[1] **MeHCH$_2$**,[2] **MeHCH$_2$-6**,[2] **MeHSiMe$_2$**,[3] **MeMeCH$_2$**,[4] **Me*i*BuCH$_2$**,[5] **MePhCH$_2$**,[6] **BrC5CH$_2$**,[7] and **BrPhCH$_2$**[1] – were prepared according to literature, and used after drying in a vacuum oven at 120 ℃ for 24 h.

1.2 Instrumental Details

^{1}H NMR spectra were collected on a Varian 400-MHz spectrometer at room temperature, using 5s delay times and 64 averaged scans. All signals were referenced to the residual solvent peak. Spectra were analyzed using MestReNova 8.1.4 [8] software package. Splitting patterns are labeled as singlet (s), doublet (d), triplet (t), or quartet (q).

Powder X-ray diffraction (PXRD) patterns were collected using a Bruker APEX II DUO CCD area-detector diffractometer operating in transmission mode. The diffractometer was equipped with a CCD detector using graphite monochromated CuK_α radiation (λ = 1.54187 Å) from an Incoatec IμS source. DUO samples were mounted either in a 0.5 mm Kapton® capillary tube, or on a standard MiTegen® mount using silicon oil, and were irradiated for 90 seconds per scan while rotating about the φ axis with three scans in the orientations of 2θ = -12, -24, and -36°, and ω = 174, 168, and 162°, respectively. The diffraction data were integrated using Apex3 v.2016.5-0 software (3-40° 2θ, with a 0.02° step size).

Analysis of PXRD patterns was conducted using Panalytical X'Pert Highscore Plus [9] software, and raw data was converted into a suitable format using the PowDLL [10] program. Experimental patterns

were compared to simulated patterns calculated from single crystal structures using Mercury [11] crystal structure viewing software and/or the Lazy Pulverix [12] package implemented in X-Seed. [13] Crystallographic Information Files containing published crystal structures were obtained from the Cambridge Structural Database (CSD) or the Crystallography Open Database (COD).

Gas adsorption analyses were conducted on a Quantachrome Instruments Autosorb-1 sorption analyzer. All samples were first activated by heating at 150 ℃ for a minimum of 18 hours, then analyzed in a 6 mm bulb cell, at 77K, with N_2 as the analysis gas. The samples were weighed 5 times, liberally applying a static gun to the cells before each weigh, in order to reduce interferences from static electricity. The average masses of the empty cell, full cell before and after outgas were used. For a full isotherm 38 points were collected in adsorption mode, with P/P_0 ranging from 0.00001 to 0.9975, and 20 points were collected in desorption mode, with P/P_0 ranging from 0.9975 to 0.05. Data were analyzed using the AS1 v. 1.55B software package and Microsoft Excel (for figure preparation). BET surface areas were calculated using adsorption points at P/P_0 = 0.007, 0.009, 0.01, 0.02, and 0.03. Total pore volume and average pore size were determined from adsorption at P/P_0 = 0.955. Pore size distribution was calculated using the Saito-Foley (SF) method using all adsorption points below P/P_0 = 0.02.

LAG-aging reactions were conducted in a 10 mL PMMA (Form-Tech Scientific) or stainless steel (InSolido Technologies) jar with one 7 mm (1.3 g) stainless steel ball bearing. The samples were milled at 30 Hz for 2 min using a Retsch MM400 ball mill. All reactions were performed by liquid-assisted grinding (LAG), where DEF was added into the jar before the start of milling with an automatic pipette. Between milling reactions the jars and ball bearings were cleaned by first milling a drop of water and ≈ 500 mg of either silica gel, or a mixture of sodium bicarbonate and solid detergent in them for 15 min, and then by sonicating in an aqueous ultrasound bath for 30-60 min. After milling, the resulting paste was transferred into a capped glass vial and left to age at room temperature. After the reaction was complete (based on PXRD), the reaction mixture was washed extensively with chloroform (according to a procedure previously shown to remove all excess template) and dried at room temperature under ambient conditions.

2. SYNTHESIS AND CHARACTERIZATION OF RHO TOPOLOGY SUBSTITUTED ZINC IMIDAZOLATES USING **MeMeCH$_2$** AS A TEMPLATE

2.1. Synthesis of 0.2**MeMeCH$_2$**@RHO-Zn$_{16}$(**Cl$_2$Im**)$_{32}$

Nanoparticulate zinc oxide (small scale: 50 mg, 0.6 mmol, large scale: 382 mg, 4.7 mmol), 4,5-dichloroimidazole (small scale: 170 mg, 1.2 mmol, large scale: 1.29 g, 9.4 mmol), **MeMeCH$_2$** (small scale: 200 mg, 0.3 mmol, large scale: 1.539 g, 2.4 mmol), and DEF (small scale: 200 μL, large scale: 1.53 mL) were milled together in a PMMA jar for 2 min at 30 Hz. The resulting dense paste was transferred to a 5 mL glass vial and left to age. Reaction progress was monitored by PXRD (Figure S5). When reaction has reached completion, as evidenced by the disappearance of ZnO X-ray reflections from the powder pattern (approx. 4 days), the sample was washed extensively with chloroform to remove excess **MeMeCH$_2$**, yielding essentially phase-pure 0.2**MeMeCH$_2$**@RHO-Zn$_{16}$(**Cl$_2$Im**)$_{32,}$ with nearly quantitative conversion based on PXRD (Figure S2). The excess template was recovered by evaporating the chloroform wash under low pressure and drying. It was reused in further experiments.

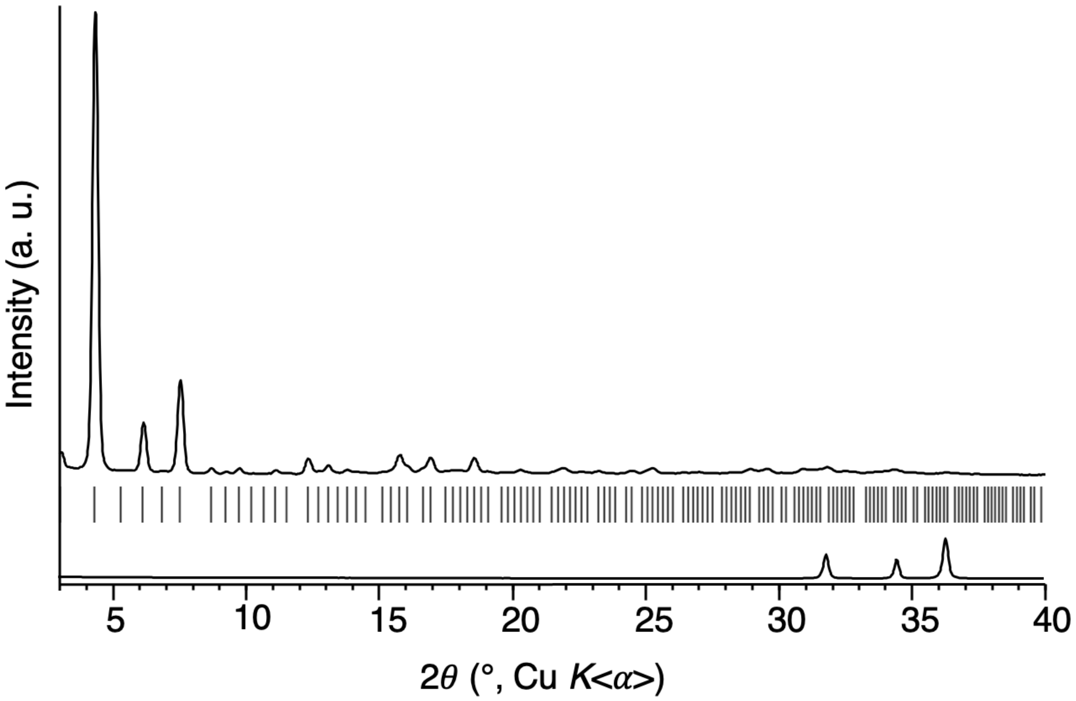

Figure S1. PXRD patterns of the washed 0.2**MeMeCH$_2$**@RHO-Zn$_{16}$(**Cl$_2$Im**)$_{32}$ product obtained by the LAG Procedure (using DEF), after 4 days (top), and zinc oxide (bottom). Tick marks denote predicted peak positions of the RHO topology Zn(**Cl$_2$Im**)$_2$ (CSD code GITVIP, ZIF-71).

The amount of enclathrated **MeMeCH₂** in the final product was calculated from the ^{1}H NMR spectrum (Figure S2) to be 1 **MeMeCH₂** per ≈ 140 4,5-dichloroimidazole molecules.

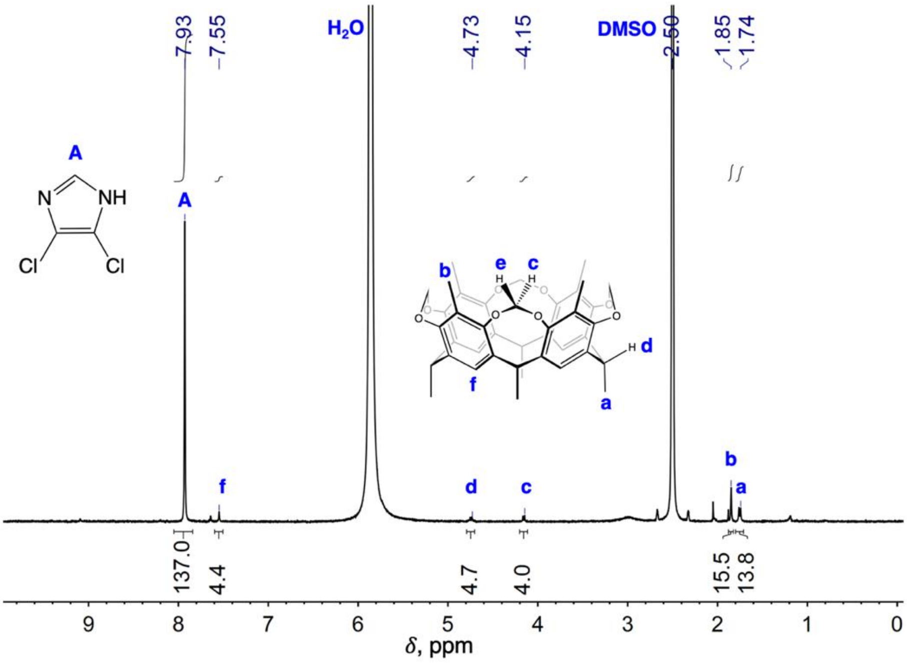

Figure S2. ^{1}H NMR spectrum of washed 0.2**MeMeCH$_2$**@RHO-Zn$_{16}$(**Cl$_2$Im**)$_{32}$ product synthesized by LAG using DEF, after aging for 4 days.

Nitrogen sorption analysis of 0.2**MeMeCH$_2$**@RHO-Zn$_{16}$(**Cl$_2$Im**)$_{32}$ (Figure S3) reveals a type I isotherm. The BET plot is linear and has a positive intercept, giving the surface area of 1140.5 m^2/g. The material maintains crystallinity and the RHO topology after activation, as evidenced by the post-sorption PXRD pattern (Figure S4).

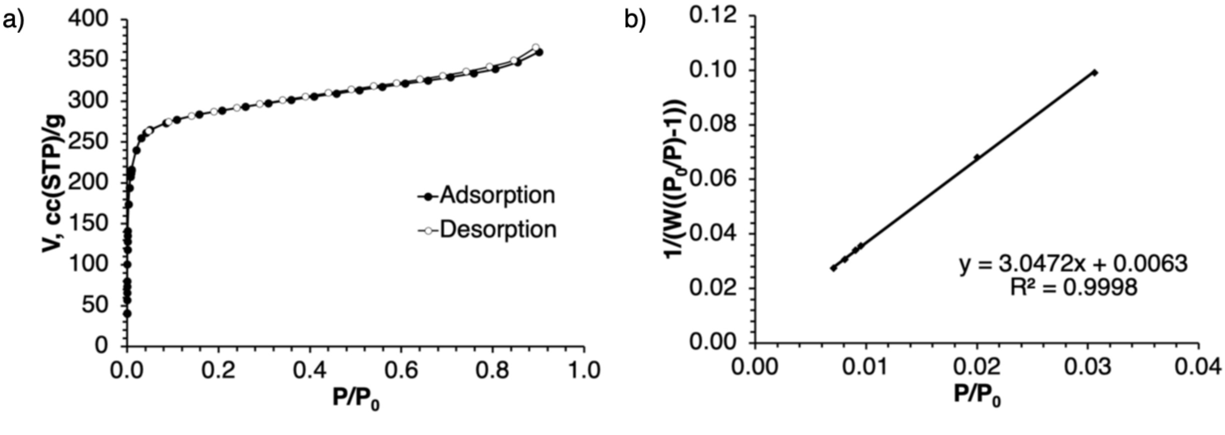

Figure S3. a) N_2 sorption isotherm at 77 K, and b) BET plot, of 0.2**MeMeCH$_2$**@RHO-Zn$_{16}$(**Cl$_2$Im**)$_{32}$ made by the LAG DEF procedure, after 4 days of aging.

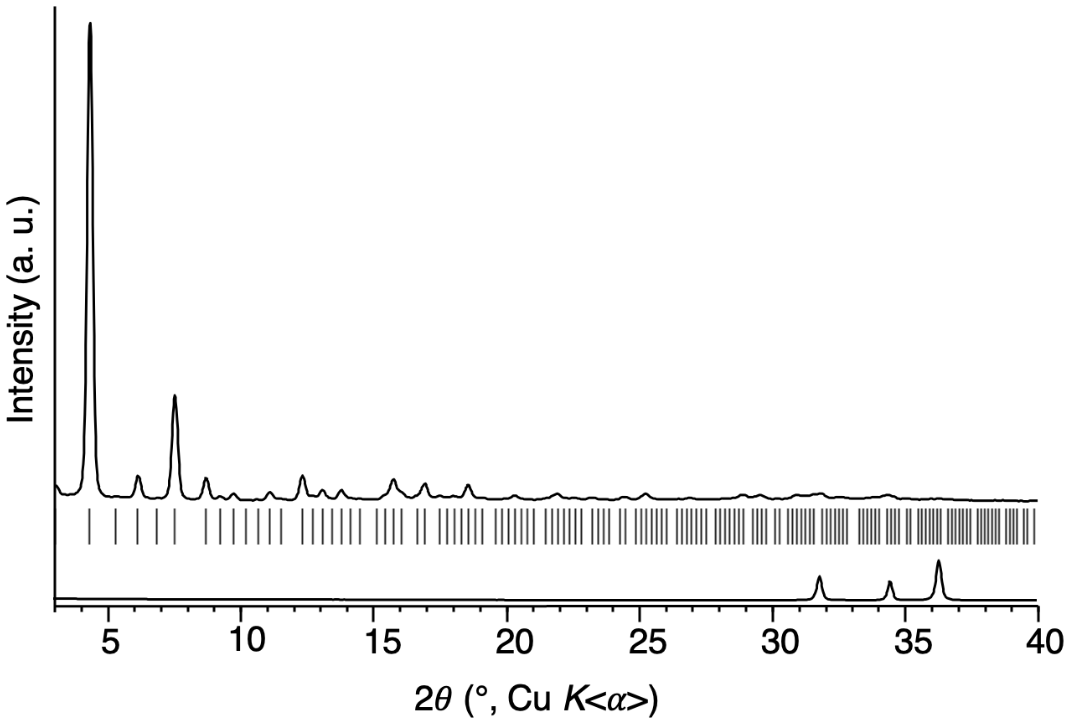

Figure S4. PXRD patterns of the washed 0.2**MeMeCH**$_2$@RHO-Zn$_{16}$(**Cl**$_2$**Im**)$_{32}$ product obtained by the LAG Procedure (using DEF), after 4 days, after activation and N_2 sorption analysis (top), and zinc oxide (bottom). Tick marks denote predicted peak positions of the RHO topology Zn(**Cl**$_2$**Im**)$_2$ (CSD code GITVIP, ZIF-71).

2.2. Control reaction and time monitoring of the LAG Procedure synthesis of 0.2**MeMeCH**$_2$@RHO-Zn$_{16}$(**Cl**$_2$**Im**)$_{32}$

As a control reaction, the procedure from section 2.1. was repeated without addition of the cavitand template. Nanoparticulate zinc oxide (50 mg, 0.6 mmol), 4,5-dichloroimidazole (170 mg, 1.2 mmol), and 60 μL of DEF were milled together in a PMMA jar for 2 min at 30 Hz. The resulting dense paste was transferred to a 5 mL glass vial and left to age. Reaction progress was monitored by PXRD (Figure S5).

Similarly to the templated reaction, the non-templated control reaction also provides an RHO topology zinc 4,5-dichloroimidazolate, namely RHO-Zn(**Cl**$_2$**Im**)$_2$ (ZIF-71). However, monitoring of the templated and non-templated reaction mixtures by PXRD over time shows that the templated reaction is significantly faster. In fact, the templated reaction after 1 day shows approximately the same conversion (based on relative amount of ZnO) as the non-templated reaction does after 35 days, and while the non-templated reaction shows a large amount of residual ZnO after 3 days, the templated reaction undergoes full conversion in that time, both on a small and large scale (Figure S5).

This shows that not only does templation using **MeMeCH$_2$** help direct the topological outcome of a mechanochemical synthesis, it also allows the reaction to proceed faster.

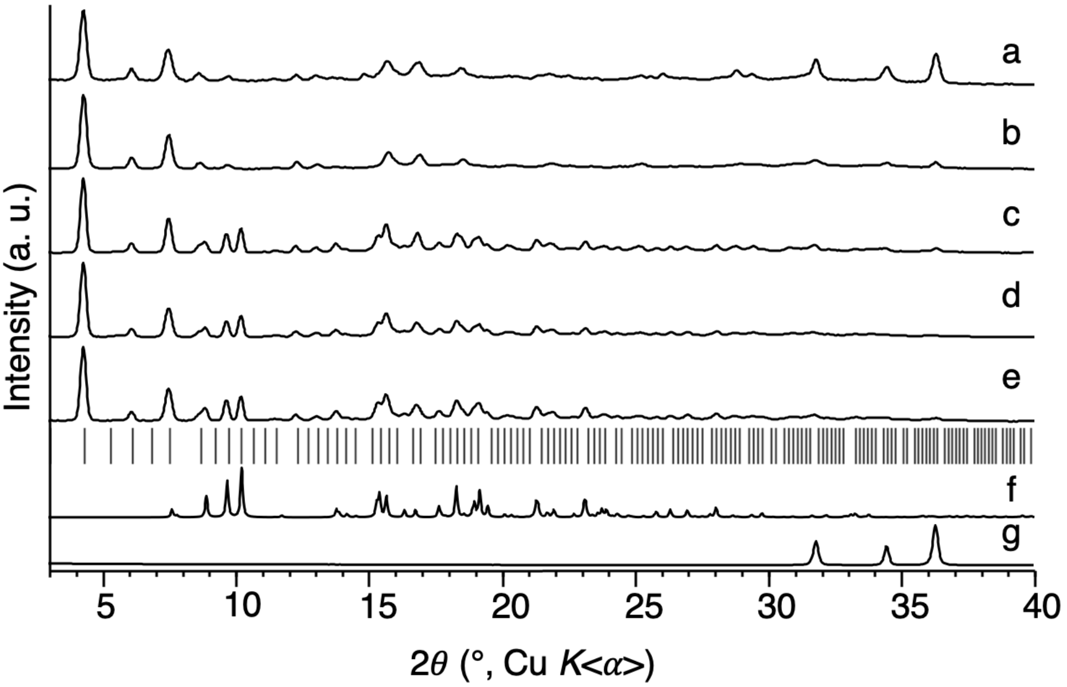

Figure S5. Comparison of PXRD patterns for: a) - b) the reaction mixture from non-templated LAG Procedure synthesis using H**Cl$_2$Im** after 3, and 35 days, respectively, c) - d) reaction mixture from templated LAG Procedure synthesis using H**Cl$_2$Im** after 1, and 3 days, respectively, e) the large scale templated LAG Procedure synthesis of 0.2**MeMeCH$_2$**@RHO-Zn$_{16}$(**Cl$_2$Im**)$_{32}$ after 4 days, f) calculated pattern of DEF@**MeMeCH$_2$**, and g) zinc oxide. Tick marks denote predicted peak positions of the RHO topology Zn(**Cl$_2$Im**)$_2$ (CSD code GITVIP, ZIF-71).

2.3. Mechanochemical synthesis of RHO-Zn**BIm**$_2$ (ZIF-11)

Nanoparticulate zinc oxide (50 mg, 0.6 mmol), benzimidazole (145 mg, 1.2 mmol), **MeMeCH$_2$** (200 mg, 0.3 mmol), and DEF (200 μL) were milled together in a PMMA jar for 2 min at 30 Hz. The resulting dense paste was transferred to a 5 mL glass vial and left to age. Reaction progress was monitored by PXRD (Figure S10). When reaction has reached completion, as evidenced by the disappearance of ZnO peaks from the powder pattern (approx. 45 days, Figure S6), the sample was washed extensively with chloroform to remove excess **MeMeCH$_2$**, yielding essentially phase-pure RHO-Zn**BIm**$_2$, with nearly quantitative conversion. The excess template was recovered by evaporating the chloroform wash under low pressure and drying. It was reused in further experiments.

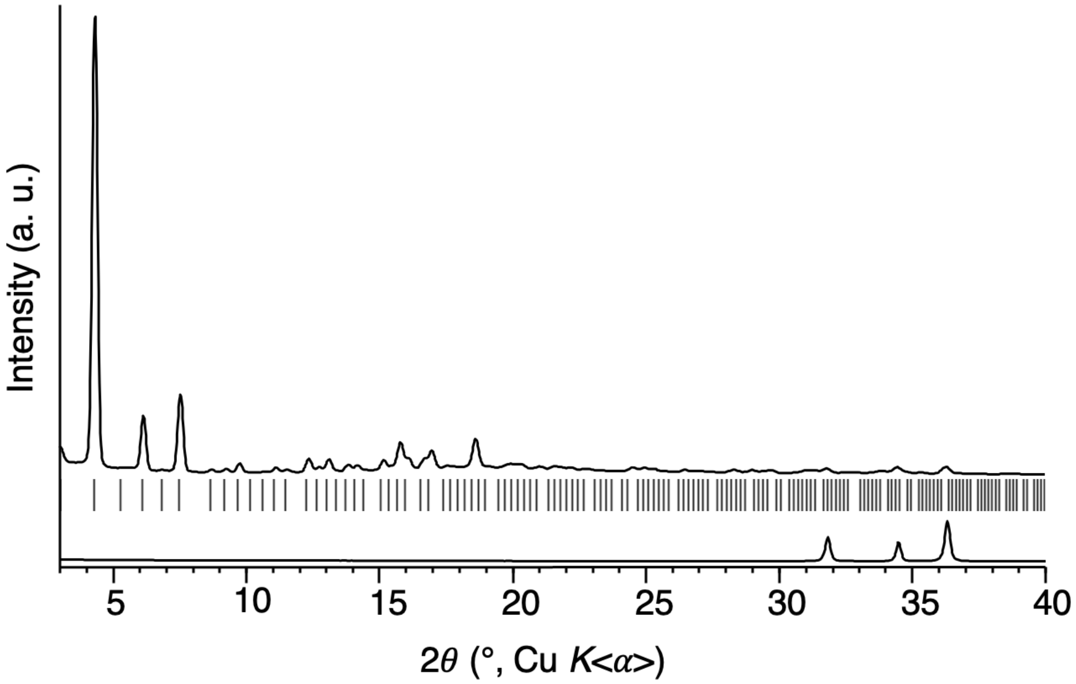

Figure S6. PXRD patterns of the washed RHO-Zn**BIm**$_2$ product obtained by the LAG Procedure (using DEF), after 45 days (top), and zinc oxide (bottom). Tick marks denote predicted peak positions of RHO-Zn**BIm**$_2$ (CSD code VEJZOA, ZIF-11).

The ^{1}H NMR spectrum shows that there is no enclathrated **MeMeCH$_2$** in the final product (Figure S7).

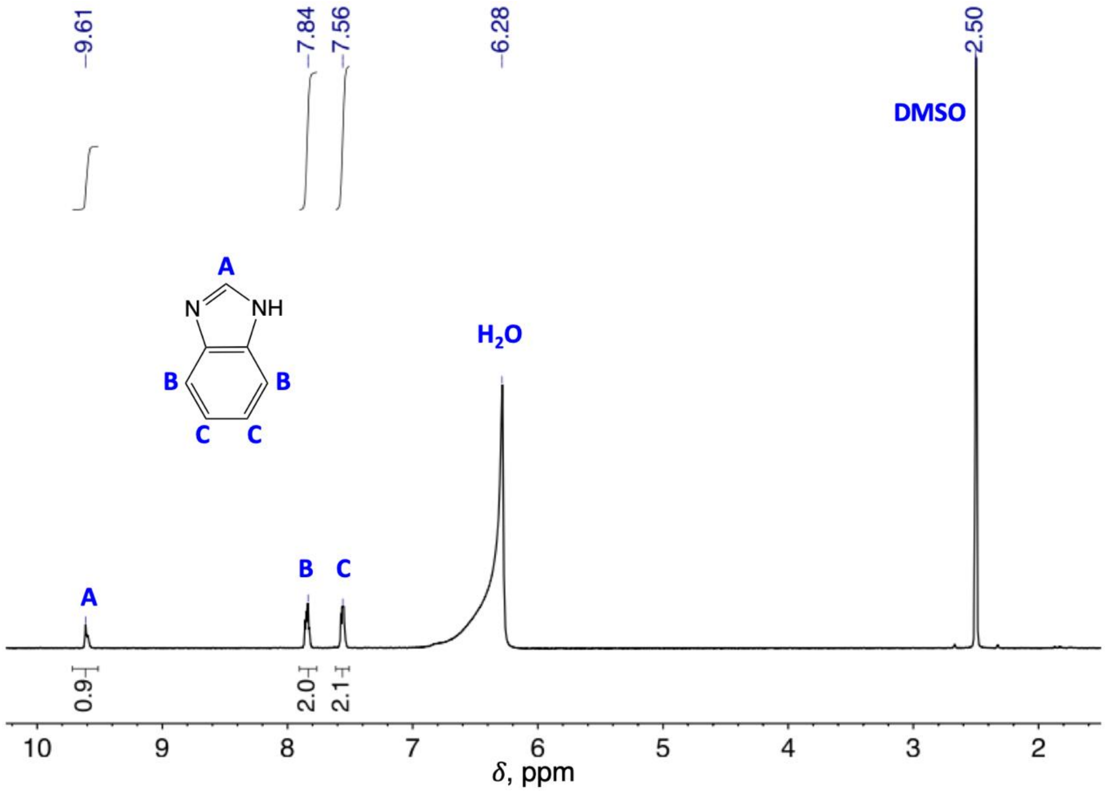

Figure S7. ^{1}H NMR spectrum of washed RHO-Zn**BIm**$_2$ product synthesized by LAG using DEF, after aging for 45 days.

Nitrogen sorption analysis of RHO-Zn**BIm**$_2$ (Figure S8) reveals a type I isotherm with slight hysteresis in the mesoporous region. The BET plot is linear and has a positive intercept, giving a surface area of 869.2 m^2/g. The material maintains crystallinity and the RHO topology after activation, as evidenced by the post-sorption PXRD pattern (Figure S9).

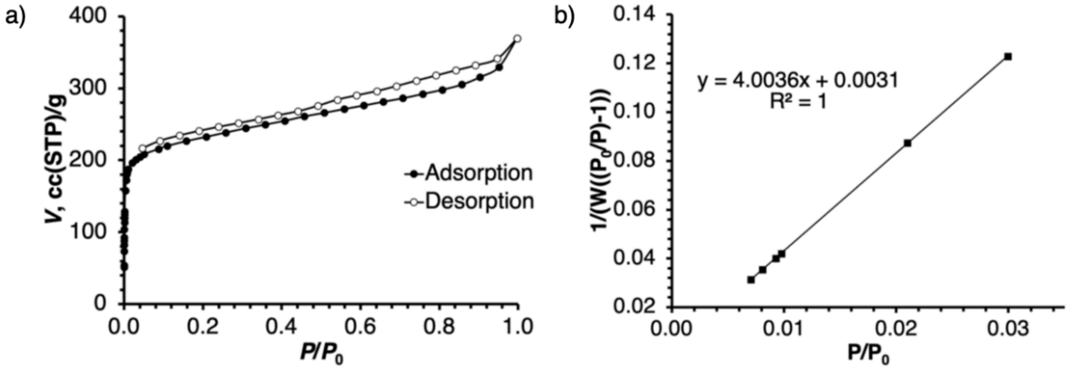

Figure S8. a) N_2 sorption isotherm at 77 K, and b) BET plot, of RHO-Zn**BIm**$_2$ made by the LAG DEF procedure, after 45 days of aging.

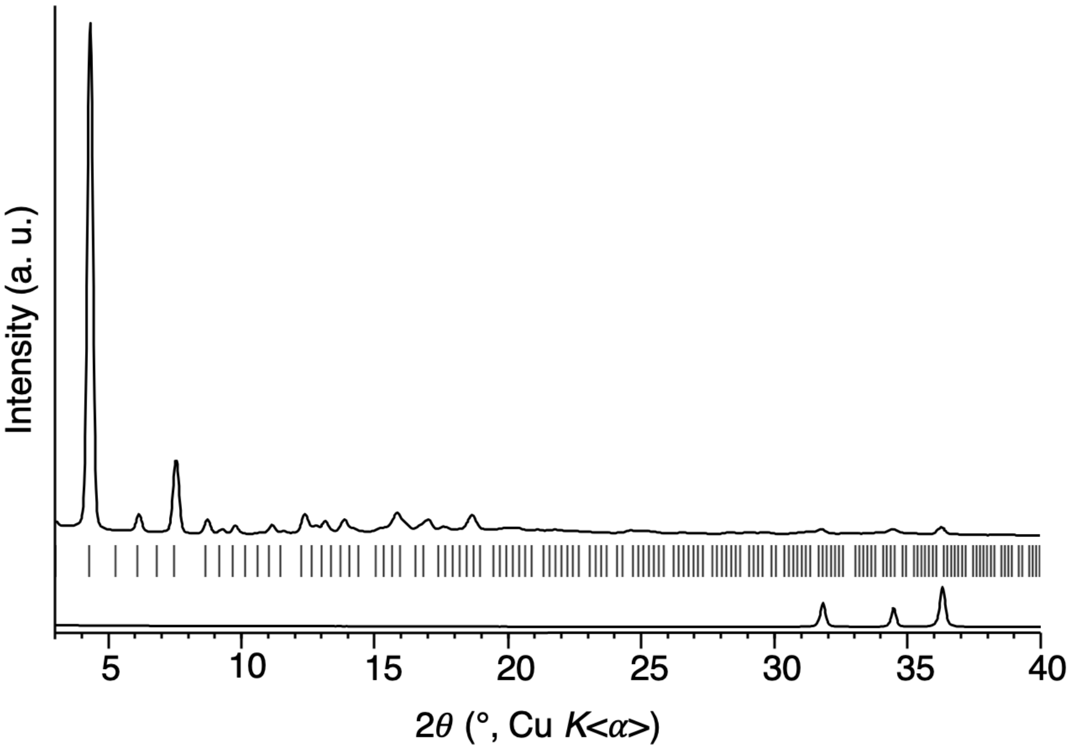

Figure S9. PXRD patterns of the washed RHO-Zn**BIm**$_2$ product obtained by the LAG Procedure (using DEF), after 45 days, after activation and N_2 sorption analysis (top), and zinc oxide (bottom). Tick marks denote predicted peak positions of the RHO topology Zn**BIm**$_2$ (CSD code VEJZOA, ZIF-11).

2.4. Control reaction and time monitoring of the LAG Procedure synthesis of RHO-Zn**BIm**$_2$

As a control reaction, the procedure from section 2.3. was repeated without addition of the cavitand template. Nanoparticulate zinc oxide (50 mg, 0.6 mmol), benzimidazole (145 mg, 1.2 mmol), and 60 μL of DEF were milled together in a PMMA jar for 2 min at 30 Hz. The resulting dense paste was transferred to a 5 mL glass vial and left to age. Reaction progress was monitored by PXRD (Figure S10).

The non-templated control reaction provides very small amounts of RHO-Zn**BIm**$_2$ after a much longer period of time, as evidenced by PXRD (Figure S10). Namely, the non-templated reaction shows no sign of an RHO topology product after 3 days, and only a very small amount after 12 days, with a very large amount of residual ZnO. Meanwhile, the templated reaction shows more conversion after 3 days than the non-templated reaction after 12 days, and is nearly quantitative after 12 days (Figure S10). This again demonstrates that **MeMeCH$_2$** significantly accelerates the synthesis of the RHO topology product.

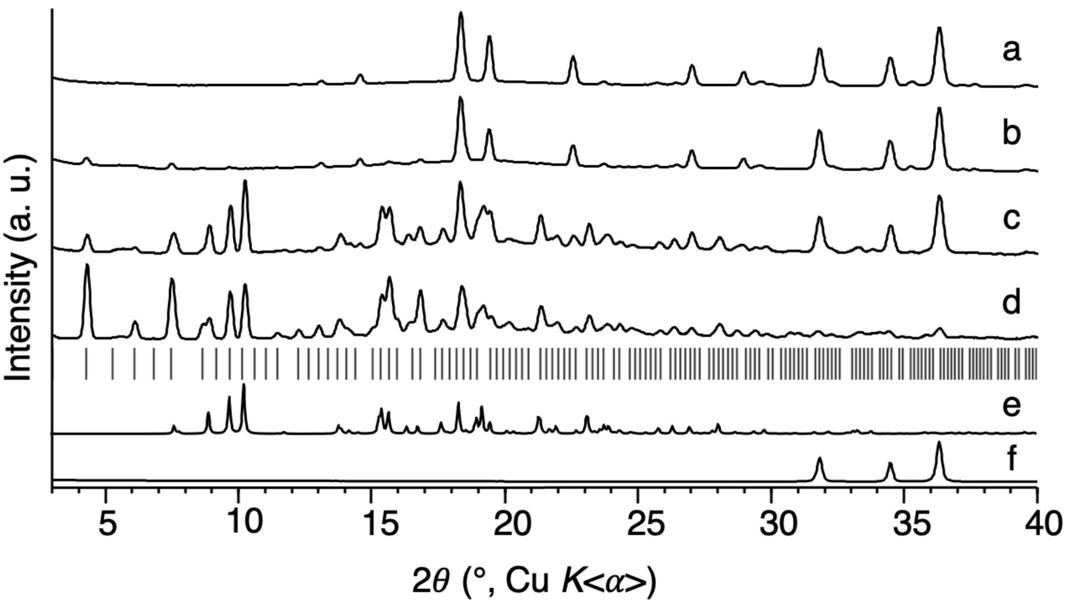

Figure S10. Comparison of PXRD patterns for the time monitoring and control reactions in the synthesis of RHO-Zn**BIm**$_2$: a) - b) the reaction mixture from non-templated LAG Procedure synthesis using H**BIm** after 3, and 12 days, respectively, c) - d) reaction mixture from templated LAG Procedure synthesis using H**BIm** after 3, and 12 days, respectively, e) calculated pattern of DEF@**MeMeCH$_2$**, and f) zinc oxide. Tick marks denote predicted peak positions of the RHO topology Zn**BIm**$_2$ (CSD code VEJZOA, ZIF-11).

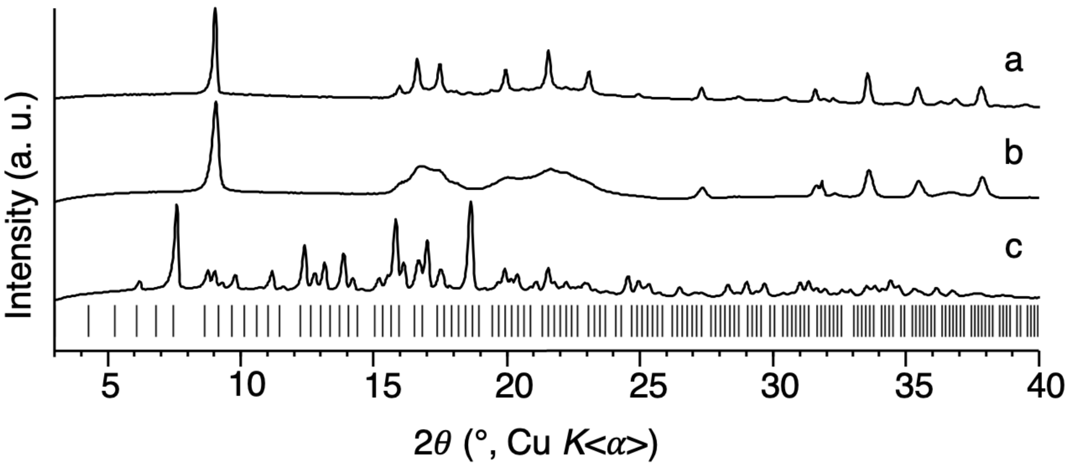

Figure S11. Comparison of PXRD patterns for products obtained from attempts to reproduce literature syntheses of RHO-Zn**BIm**$_2$: a) the original reported synthesis,[14] and b) – c) toluene templated synthesis [15] (2 attempts). Tick marks denote predicted peak positions of the RHO topology Zn**BIm**$_2$ (CSD code VEJZOA, ZIF-11).

2.5. Templated mechanochemical experiments using **MeMeCH$_2$** and other ligands

Nanoparticulate zinc oxide (50 mg, 0.6 mmol), **MeMeCH$_2$** (200 mg, 0.3 mmol), and DEF (200 μL) were milled together with 1.2 mmol of different imidazole derivatives: 5-nitrobenzimidazole (205 mg), 5,6-dimethylbenzimidazole (185 mg), or theophylline (228 mg) in a PMMA jar for 2 min at 30 Hz. The resulting dense pastes were transferred to a 5 mL glass vial and left to age. Reaction progress was monitored by PXRD (Figure S12). None of the reactions appeared to show any trace of an RHO topology product, based on PXRD. The reaction using theophylline provided the known zinc theophylline dihydrate after a year, while the reactions using 5-nitrobenzimidazole and 5,6-dimethylbenzimidazole showed almost no conversion after 37 days, as evidenced by PXRD.

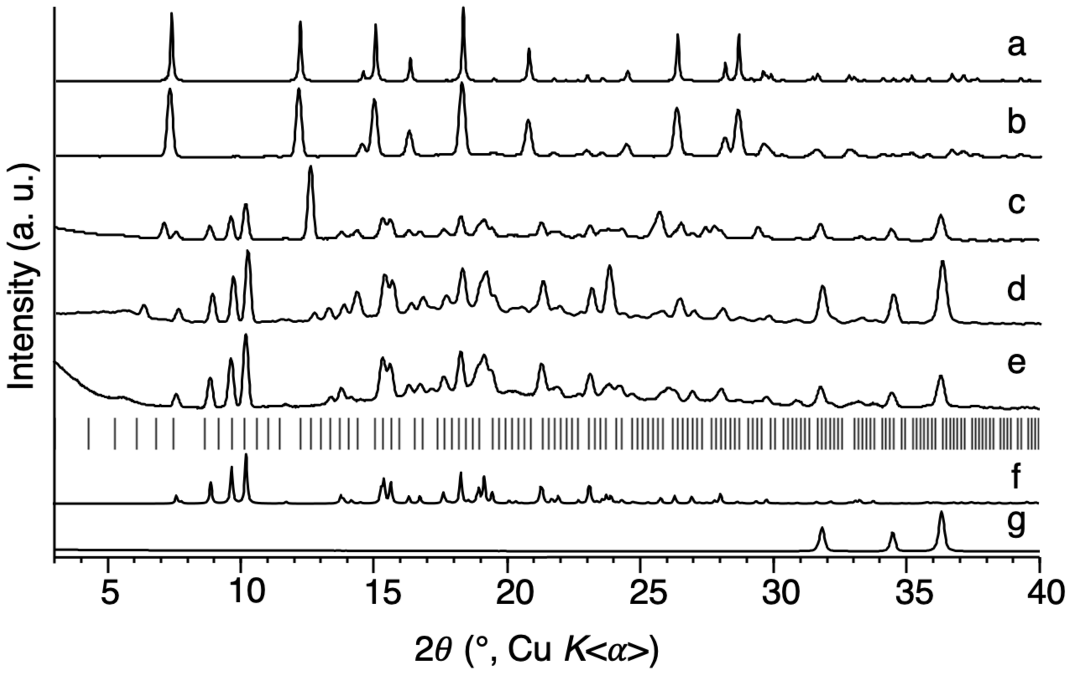

Figure S12. Comparison of PXRD patterns for the templated solid-state reactions using theophylline, 5,6-dimethylbenzimidazole, and 5-nitrobenzimidazole: a) predicted peak positions of zinc theophylline dihydrate (CSD code CIBLIK), b) – c) the reaction mixture from the **MeMeCH$_2$** templated LAG Procedure synthesis using theophylline after 1 year (washed), and 22 days, respectively, d) reaction mixture from the **MeMeCH$_2$** templated LAG Procedure synthesis using 5,6-dimethylbenzimidazole after 37 days, e) reaction mixture from the **MeMeCH$_2$** templated LAG Procedure synthesis using 5-nitrobenzimidazole after 37 days, f) calculated pattern of DEF@**MeMeCH$_2$**, and g) zinc oxide. Tick marks denote predicted peak positions of the RHO topology Zn**BIm$_2$** (CSD code VEJZOA, ZIF-11).

3. SYNTHESIS AND CHARACTERIZATION OF RHO TOPOLOGY ZINC IMIDAZOLATES USING OTHER CAVITAND TEMPLATES

To further explore the scope of the templation effect, we employed a variety of cavitands other than **MeMeCH**$_2$ in the mechanochemical synthesis of zinc imidazolates. We varied the top rim substituent, the bottom rim substituent, the top rim linker, as well as the number of repeating units in the cavitand. In a typical LAG-aging experiment, nanoparticulate zinc oxide, imidazole, the template, and DEF were mixed in a 1 : 2 : 0.5 : 4 molar ratio, and milled in a PMMA jar for 2 min at 30 Hz. The resulting dense paste was transferred to a 5 mL glass vial and left to age. Reaction progress was monitored by PXRD. When reaction has reached completion, as evidenced by the disappearance of ZnO peaks from the powder pattern, the sample was washed extensively with chloroform to remove excess cavitand.

3.1. LAG-aging reaction using **HPhCH**$_2$ as template

LAG-aging experiment was performed using nanoparticulate zinc oxide (48 mg, 0.6 mmol), imidazole (82 mg, 1.2 mmol), **HPhCH**$_2$ (246 mg, 0.3 mmol), and DEF (200 μL). The mixture was washed after 12 days, giving 0.8**HPhCH**$_2$@RHO-Zn_{16}**Im**$_{32}$, as confirmed by NMR and PXRD (Figure S14, S13). Interestingly, the PXRD pattern of 0.8**HPhCH**$_2$@RHO-Zn_{16}**Im**$_{32}$ shows very different peak intensities compared to the other RHO materials (Figure S14). Namely, the (100) peak (at ~3° 2θ) that is normally very small in PXRD patterns of RHO topology zeolitic imidazolate frameworks is very intense in this pattern, while the (110) peak – which is normally the dominant one – is much smaller. In addition, there is significant broadening of several peaks, while the (100) peak is still sharp. Both effects could be due to the large quantity **HPhCH**$_2$ trapped inside the double-8-rings of the material, causing the phenyl feet to protrude into the LTA cage. The electron density in the cage will affect peak intensities, while steric hindrance could cause twisting and breathing of the framework itself, resulting in peak broadening.

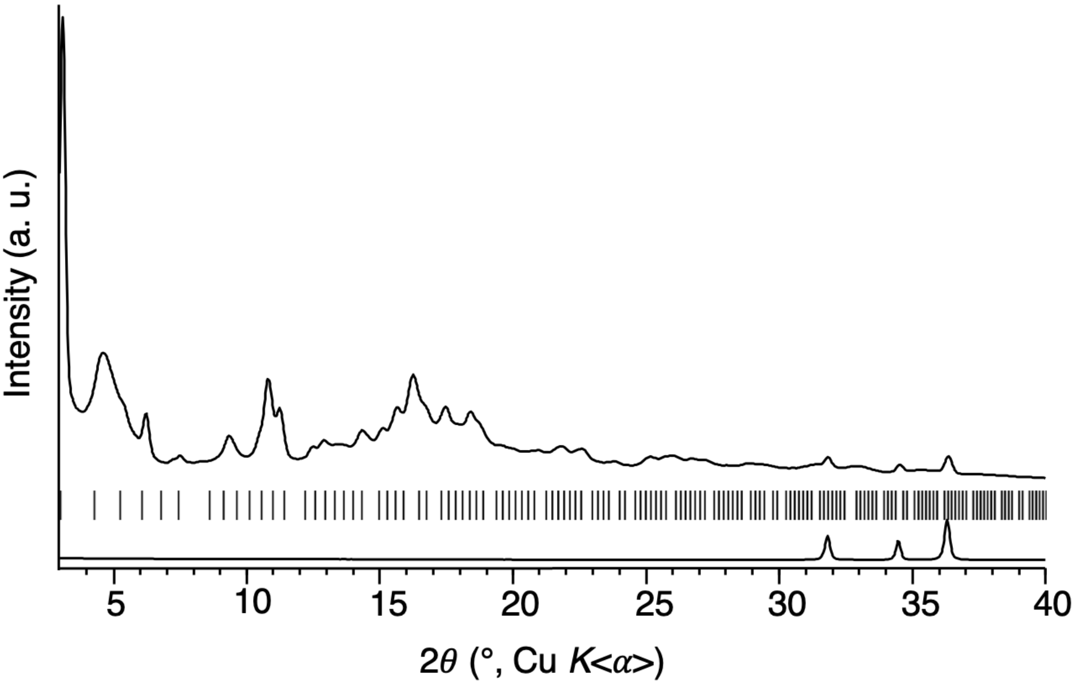

Figure S13. Comparison of PXRD patterns for 0.8**HPhCH$_2$**@RHO-Zn$_{16}$**Im**$_{32}$ synthesized by the LAG-aging procedure using **HPhCH$_2$** and washed after 12 days (top), and zinc oxide (bottom). Tick marks denote predicted peak positions of a cubic unit cell, *Pm*-3*m*, *a* = 28.907(2) Å, corresponding to *x***MeMeCH$_2$**@RHO-Zn$_{16}$**Im**$_{32}$.

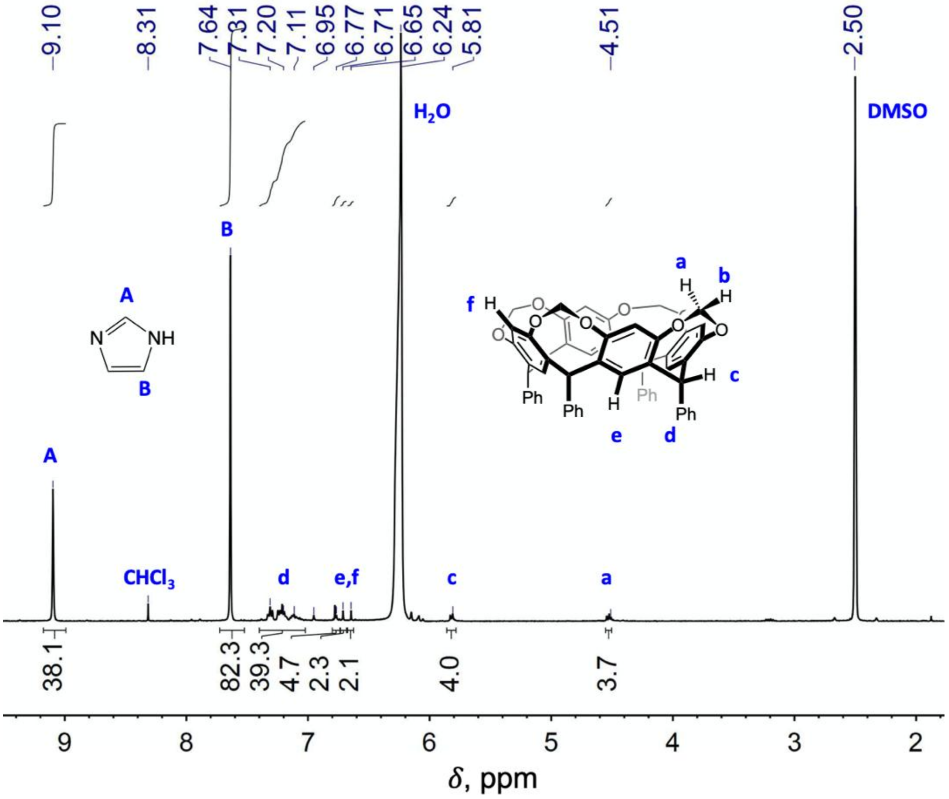

Figure S14. ^{1}H NMR spectrum (1 mL DMSO-d6, 50μL DCl) of 0.8**HPhCH$_2$**@RHO-Zn$_{16}$**Im**$_{32}$, synthesized by the LAG-aging procedure using **HPhCH$_2$** and washed after 12 days.

3.2. LAG-aging reaction using **H(4MePh)CH$_2$** as template

LAG-aging experiment was performed using nanoparticulate zinc oxide (17 mg, 0.2 mmol), imidazole (28 mg, 0.4 mmol), **H(4MePh)CH$_2$** (90 mg, 0.1 mmol), and DEF (70 μL). The mixture was washed after 11 days, resulting in a mixture of products (major product is **cag**-Zn**Im**$_2$) that does not contain any RHO topology product, as confirmed by PXRD (Figure S15).

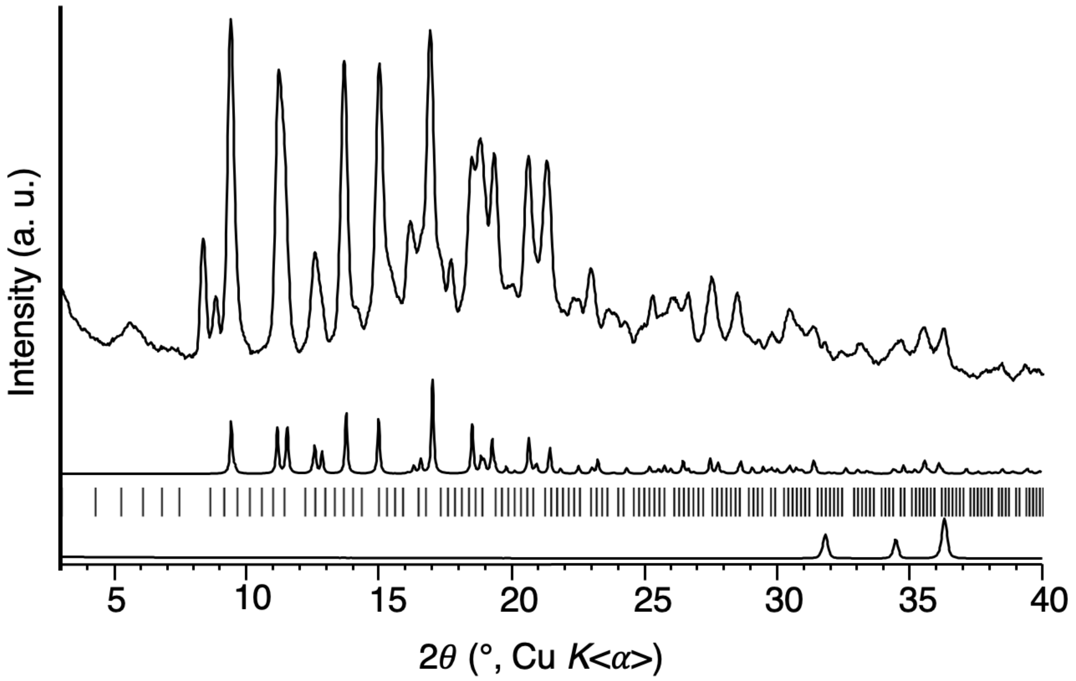

Figure S15. Comparison of PXRD patterns for **cag**-Zn**Im**$_2$, synthesized by the LAG-aging procedure using **H(4MePh)CH$_2$** and washed after 11 days (top) and zinc oxide (bottom), and the predicted PXRD pattern of **cag**-Zn**Im**$_2$ (middle, CSD code VEJYUF). Tick marks denote predicted peak positions of a cubic unit cell, *Pm*-3*m*, *a* = 28.907(2) Å, corresponding to *x***MeMeCH$_2$**@RHO-Zn$_{16}$**Im**$_{32}$.

3.3. LAG-aging reaction using **MeHCH$_2$** as template

LAG-aging experiment was performed using nanoparticulate zinc oxide (40 mg, 0.5 mmol), imidazole (68 mg, 1.0 mmol), **MeHCH$_2$** (143 mg, 0.25 mmol), and DEF (145 μL). The mixture was washed after 16 days, giving 1.6**MeHCH$_2$**@RHO-Zn$_{16}$**Im**$_{32}$, as confirmed by NMR and PXRD (Figure S17, S16)).

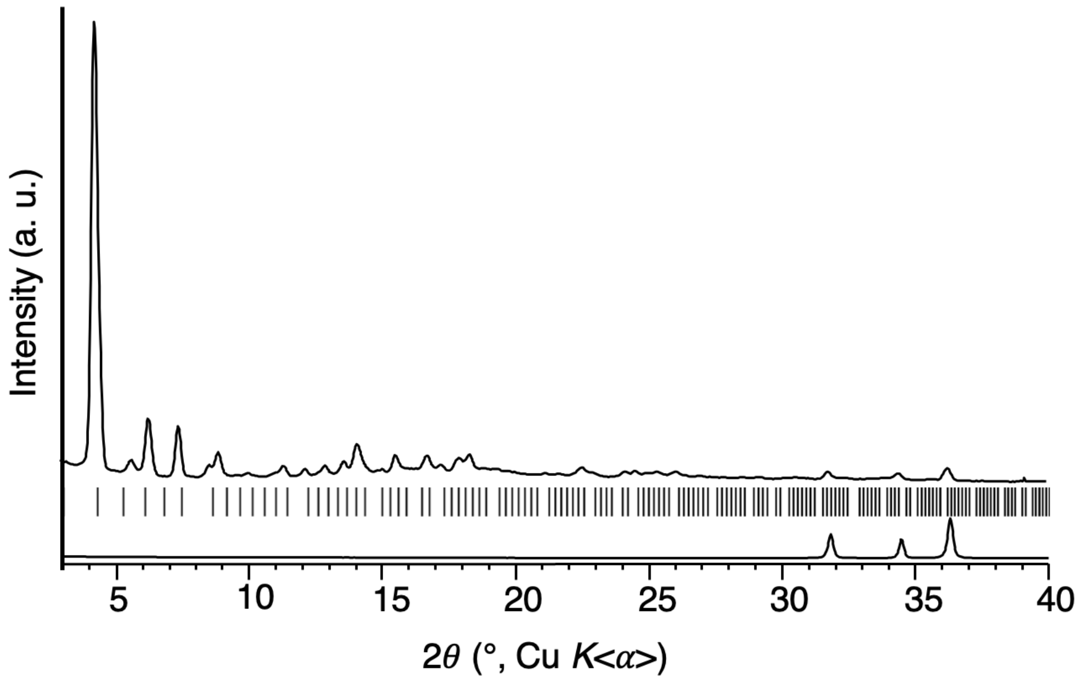

Figure S16. PXRD patterns of 1.6**MeHCH$_2$**@RHO-Zn$_{16}$**Im**$_{32}$ synthesized by the LAG-aging procedure using **MeHCH$_2$** and washed after 16 days (top), and zinc oxide (bottom). Tick marks denote predicted peak positions of a cubic unit cell, *Pm*-3*m*, *a* = 28.907(2) Å, corresponding to *x***MeMeCH$_2$**@RHO-Zn$_{16}$**Im**$_{32}$.

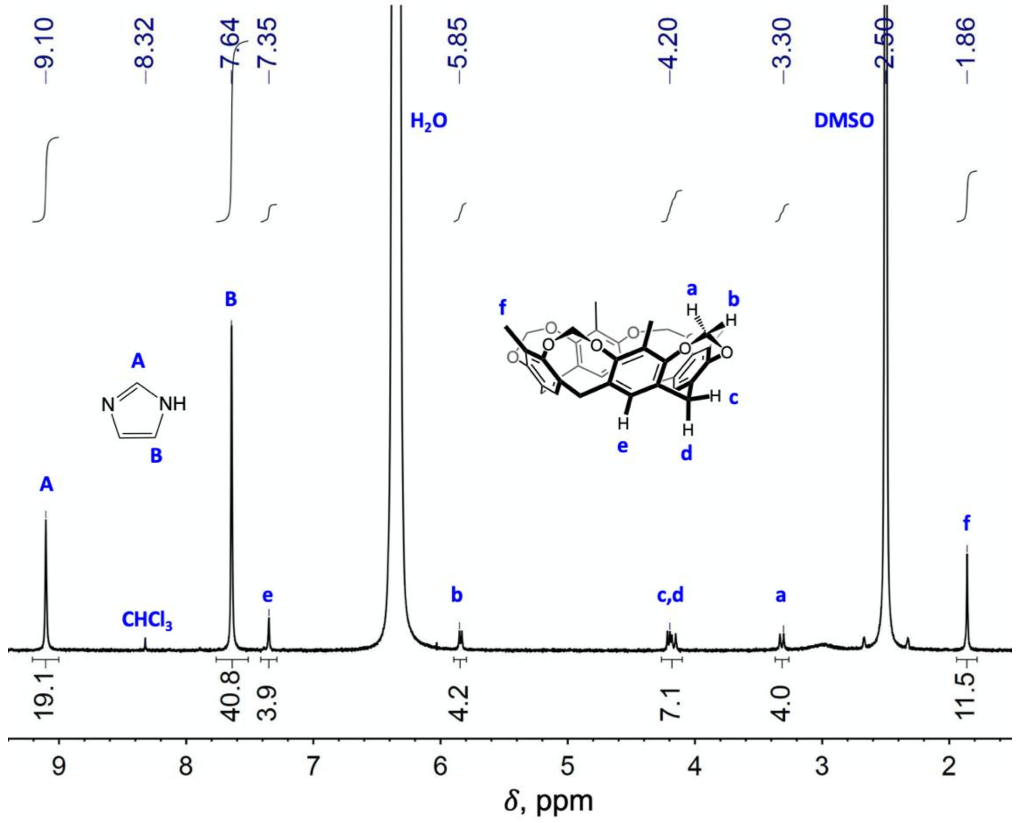

Figure S17. ^{1}H NMR spectrum (1 mL DMSO-*d*$_6$, 50μL DCl) of 1.6**MeHCH$_2$**@RHO-Zn$_{16}$**Im**$_{32}$, synthesized by the LAG-aging procedure using **MeHCH$_2$** and washed after 16 days

3.4. LAG-aging reaction using **MeHCH$_2$-6** as template

LAG-aging experiment was performed using nanoparticulate zinc oxide (25 mg, 0.3 mmol), imidazole (42 mg, 0.6 mmol), **MeHCH$_2$-6** (136 mg, 0.15 mmol), and DEF (100 μL). The mixture was washed after 5 days, giving **MeHCH$_2$-6@**RHO-Zn$_{16}$**Im**$_{32}$ as confirmed by NMR and PXRD (Figure S19, S18).

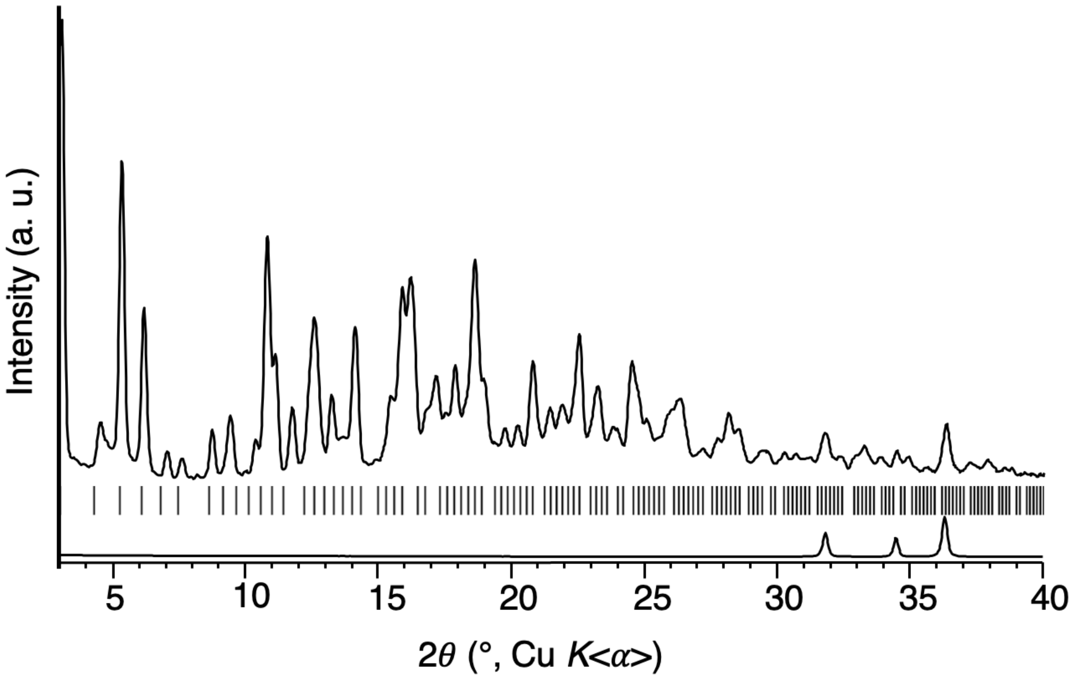

Figure S18. PXRD patterns of 5.8**MeHCH$_2$-6**@RHO-Zn$_{16}$**Im**$_{32}$prepared by the LAG-aging procedure using **MeHCH$_2$-6,** washed after 5 days (top), and zinc oxide (bottom). Tick marks denote predicted peak positions of a cubic unit cell, *Pm*-3*m*, *a* = 28.907(2) Å, corresponding to *x***MeMeCH$_2$**@RHO-Zn$_{16}$**Im**$_{32}$.

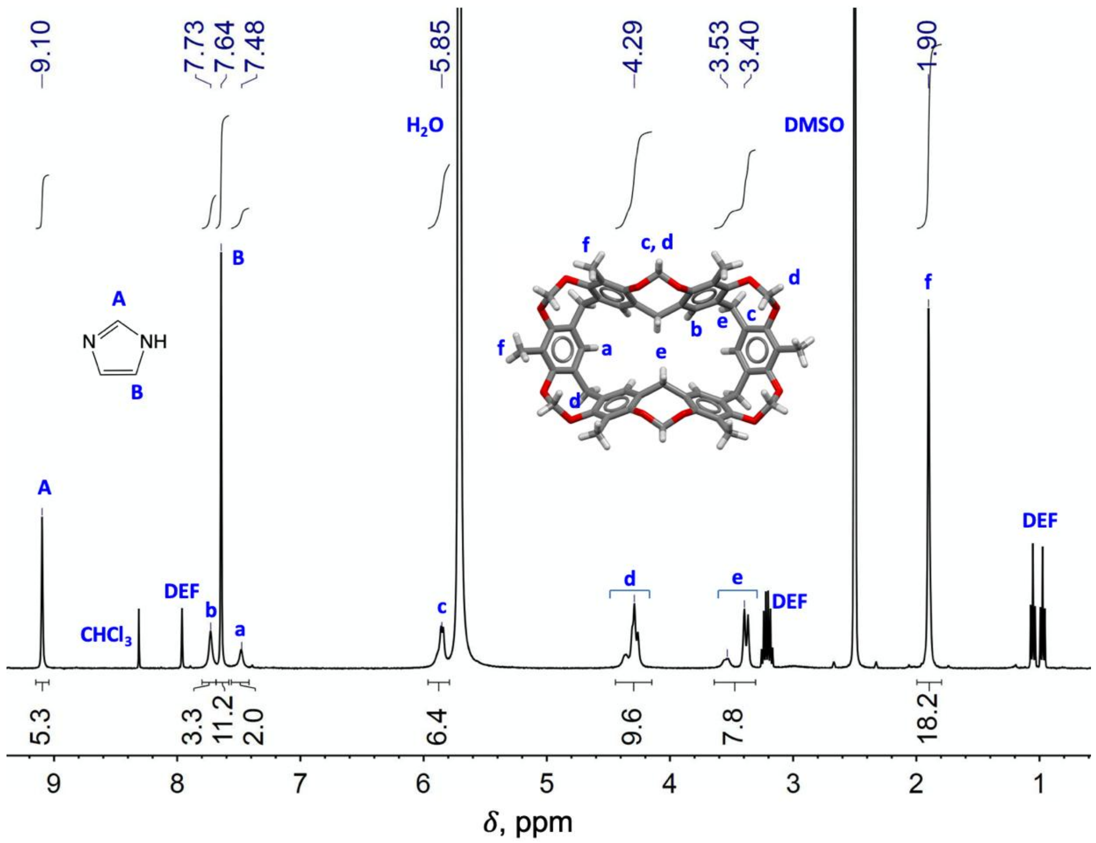

Figure S19. NMR spectrum (1 mL DMSO-d_6, 50μL DCl) of 5.8**MeHCH₂-6**@RHO-Zn_{16}**Im**$_{32}$, synthesized by the LAG-aging procedure using **MeHCH₂-6** and washed after 5 days

3.5. LAG-aging reaction using **MeHSiMe₂** as template

LAG-aging experiment was performed using nanoparticulate zinc oxide (96 mg,1.2 mmol), imidazole (163 mg, 2.4 mmol), **MeHSiMe₂** (450 mg, 0.6 mmol), and DEF (400 μL). The mixture was washed after 11 days, giving a mixture of products, with the major being **cag**-Zn**Im**$_2$, and containing no RHO topology product, as confirmed by PXRD (Figure S20).

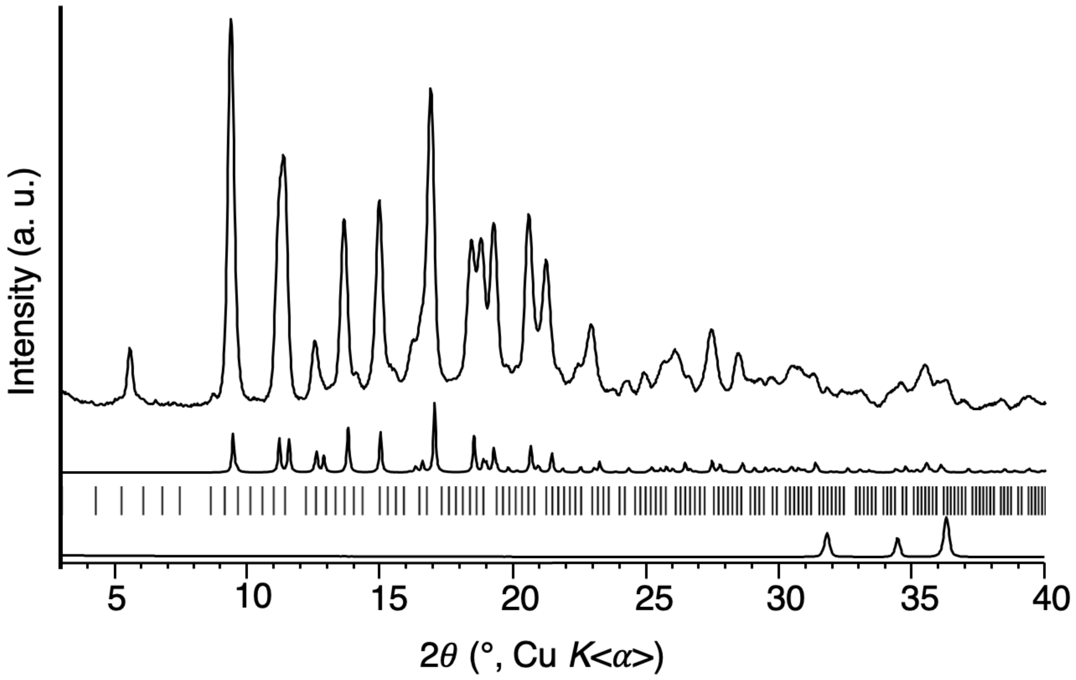

Figure S20. PXRD patterns of **cag**-Zn**Im**$_2$, synthesized by the LAG-aging procedure using **MeHSiMe$_2$**, washed after 11 days (top), and zinc oxide (bottom), and the predicted PXRD pattern of **cag**-Zn**Im**$_2$ (middle, CSD code VEJYUF). Tick marks denote predicted peak positions of a cubic unit cell, *Pm*-3*m*, *a* = 28.907(2) Å, corresponding to *x***MeMeCH$_2$**@RHO-Zn$_{16}$**Im**$_{32}$.

3.6. LAG-aging reaction using **Me*i*BuCH$_2$** as template

LAG-aging experiment was performed using nanoparticulate zinc oxide (50 mg, 0.6 mmol), imidazole (86 mg, 1.2 mmol), **Me*i*BuCH$_2$** (252 mg, 0.3 mmol), and DEF (250 μL). The mixture was washed after 16 days, giving 0.3**Me*i*BuCH$_2$**@RHO-Zn$_{16}$**Im**$_{32}$, as confirmed by NMR and PXRD (Figure S22, S21).

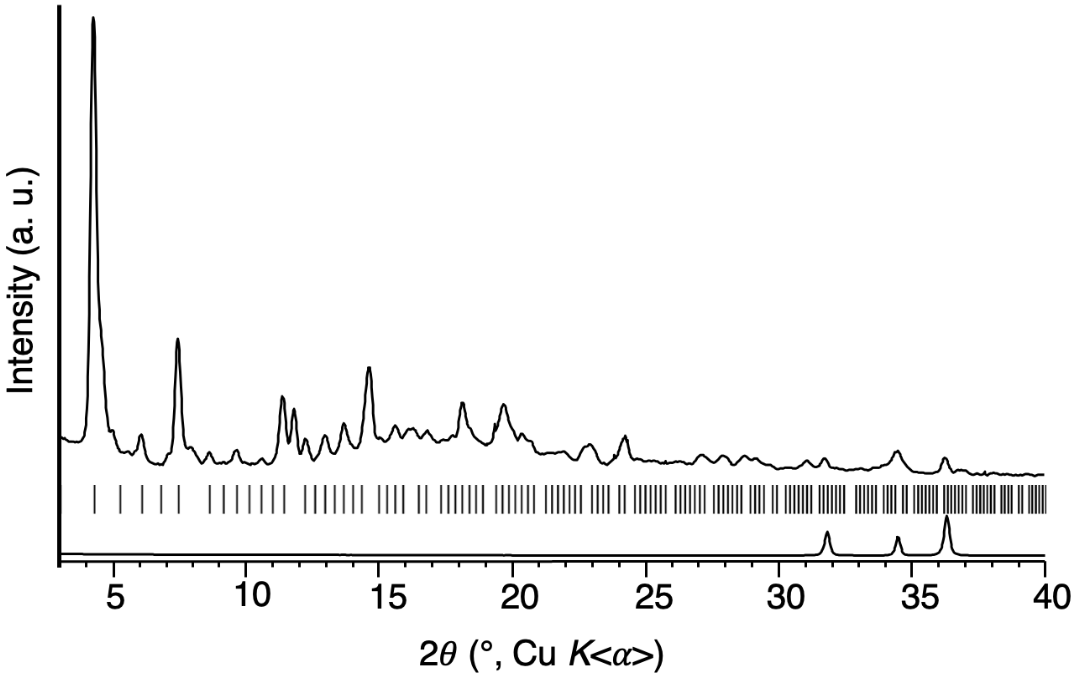

Figure S21. PXRD patterns of 0.3**Me*i*BuCH$_2$**@RHO-Zn$_{16}$**Im**$_{32}$, synthesized by the LAG-aging procedure using **Me*i*BuCH$_2$** and washed after 16 days (top), and zinc oxide (bottom). Tick marks denote predicted peak positions of a cubic unit cell, *Pm*-3*m*, *a* = 28.907(2) Å, corresponding to *x***MeMeCH$_2$**@RHO-Zn$_{16}$**Im**$_{32}$.

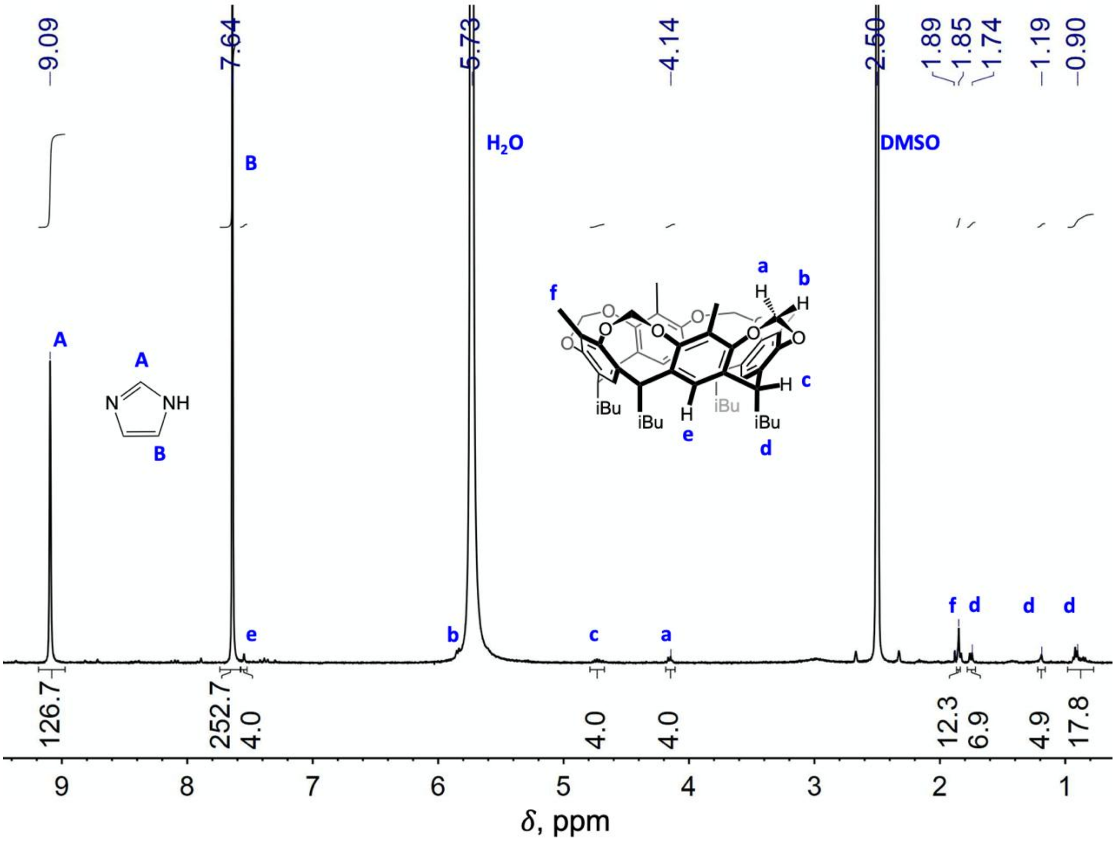

Figure S22. ^{1}H NMR spectrum (1 mL DMSO-d_6, 50μL DCl) of 0.3**Me*i*BuCH$_2$**@RHO-Zn$_{16}$**Im**$_{32}$, synthesized by the LAG-aging procedure using **Me*i*BuCH$_2$** and washed after 16 days

3.7. LAG-aging reaction using **MePhCH2** as template

LAG-aging experiment was performed using nanoparticulate zinc oxide (25 mg, 0.3 mmol), imidazole (42 mg, 0.6 mmol), **MePhCH2** (87% **rctt** : 13% **rccc**, 139 mg, 0.15 mmol), and DEF (100 μL). The reaction product was washed after 5 days, giving a mixture of x**MePhCH2**@RHO-Zn_{16}**Im**$_{32}$, and **nog**-Zn**Im**$_2$, as confirmed by PXRD (Figure S24).

NMR analysis of the starting **MePhCH2** cavitand shows that it is a mixture of the **rctt** and **rccc** isomers, in the ratio of 87:13. Similarly, the NMR analysis of the washed product (Figure S24) shows that the encapsulated cavitand exactly mimics that stereochemical ratio, with the total cavitand : imidazole ratio being 1:17.3. Unfortunately, calculating the incorporation of cavitand into the RHO topology zinc imidazolate is impossible, as the relative quantities of the **nog** and RHO topology products cannot be determined.

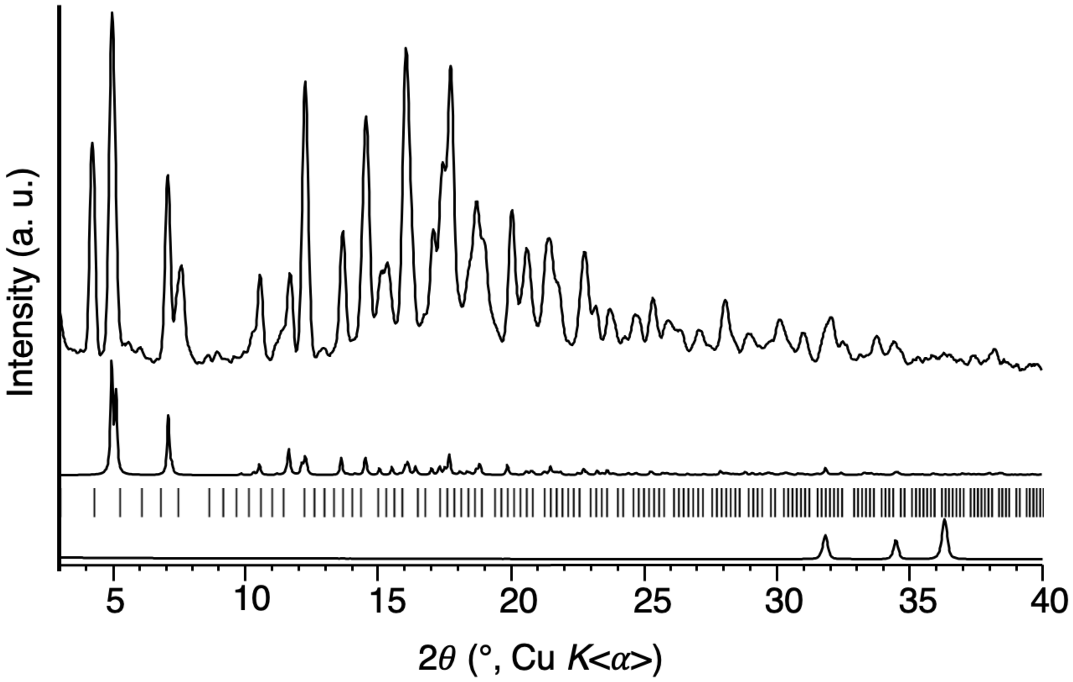

Figure S23. PXRD patterns of the product of the LAG-aging procedure using **MePhCH2**, washed after 5 days (top), and zinc oxide (bottom), and the predicted PXRD pattern of **nog**-Zn**Im**$_2$ (middle, CSD code: HIFWAV). Tick marks denote predicted peak positions of a cubic unit cell, *Pm*-3*m*, *a* = 28.907(2) Å, corresponding to *x***MeMeCH2**@RHO-Zn_{16}**Im**$_{32}$.

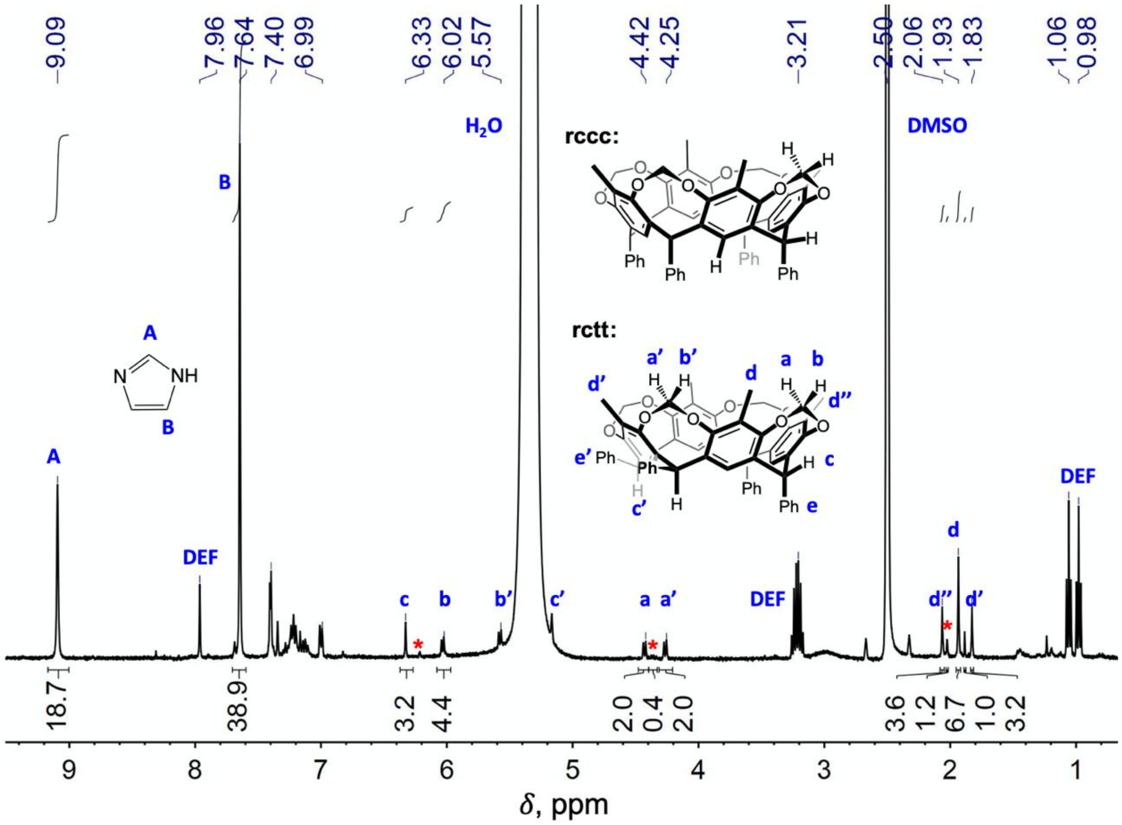

Figure S24. ^{1}H NMR spectrum (1 mL DMSO-d_6, 50μL DCl) of the product of the LAG-aging procedure using **MePhCH$_2$**, washed after 5 days. Red stars denote signals of the **rccc** stereoisomer.

3.8. LAG-aging reaction using **BrC5CH$_2$** as template

LAG-aging experiment was performed using nanoparticulate zinc oxide (25 mg, 0.3 mmol), imidazole (42 mg, 0.6 mmol), **BrC5CH$_2$** (133 mg, 0.15 mmol), and DEF (100 μL). The mixture was washed after 5 days, giving 0.2**BrC5CH$_2$**@RHO-Zn_{16}**Im**$_{32}$, as confirmed by NMR and PXRD (Figure S26, S25).

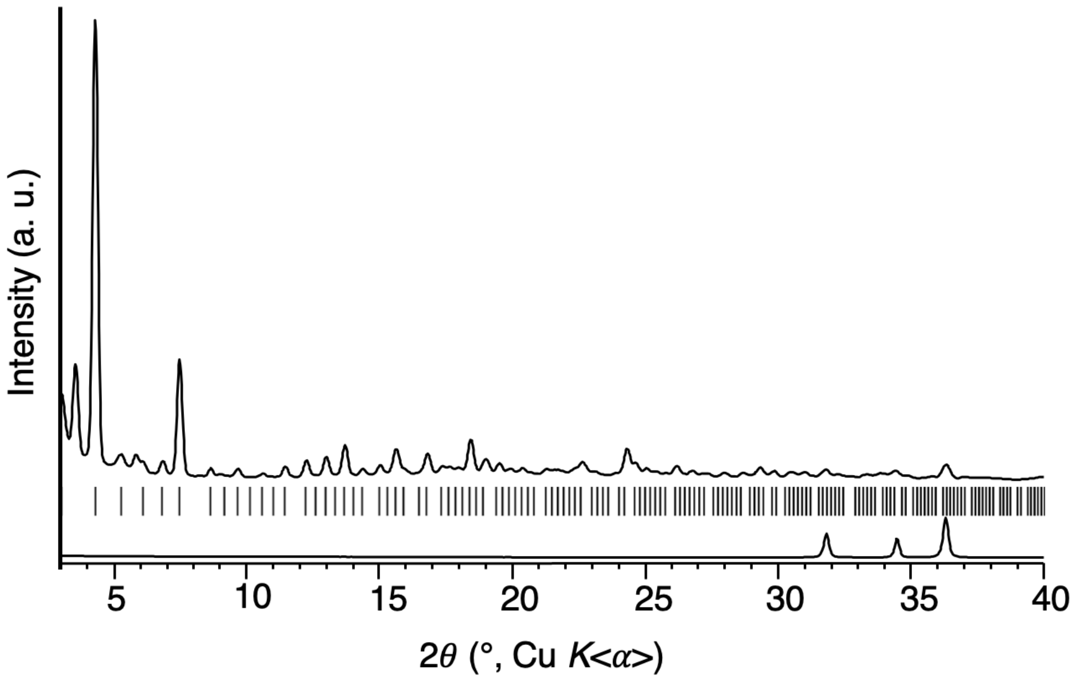

Figure S25. PXRD patterns of 0.2**BrC5CH$_2$**@RHO-Zn$_{16}$**Im**$_{32}$, synthesized by the LAG-aging procedure using **BrC5CH$_2$** and washed after 5 days (top), and zinc oxide (bottom). Tick marks denote predicted peak positions of a cubic unit cell, *Pm*-3*m*, *a* = 28.907(2) Å, corresponding to *x***MeMeCH$_2$**@RHO-Zn$_{16}$**Im**$_{32}$.

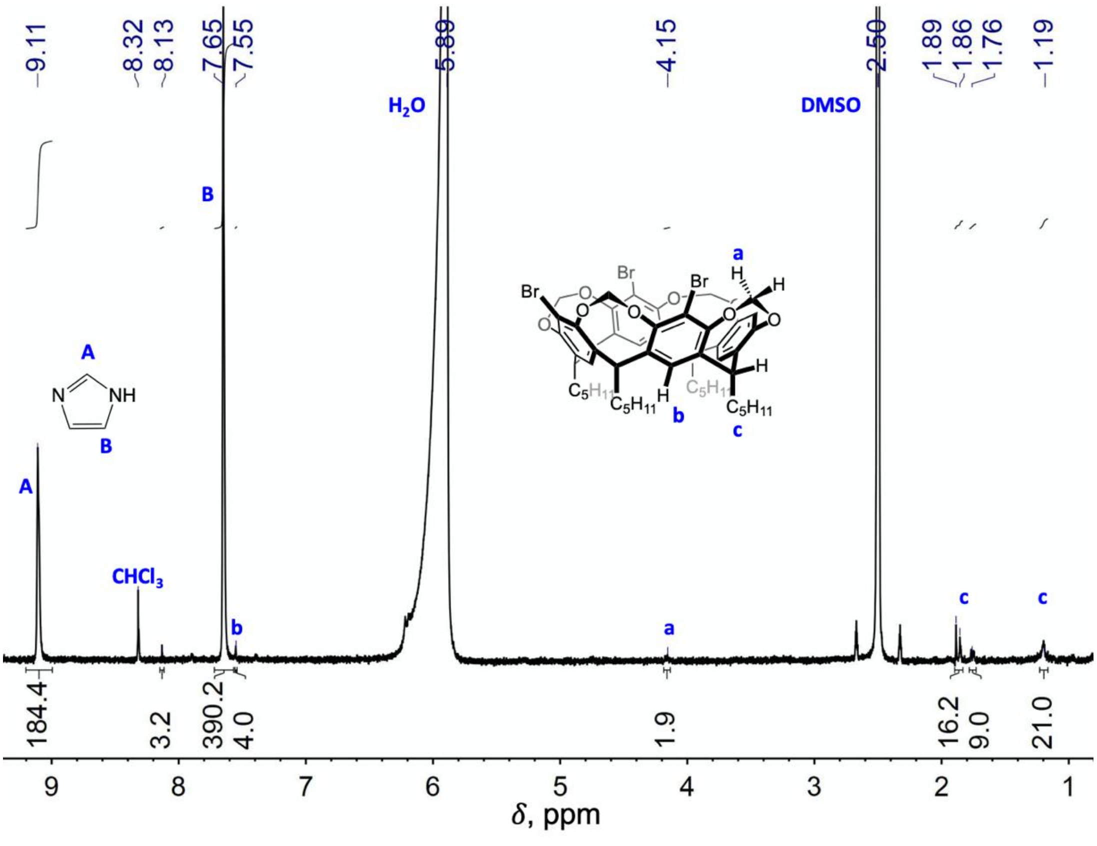

Figure S26. ^{1}H NMR spectrum (1 mL DMSO-d_6, 50μL DCl) of 0.2**BrC5CH$_2$**@RHO-Zn$_{16}$**Im**$_{32}$, synthesized by the LAG-aging procedure using **BrC5CH$_2$** and washed after 5 days

3.9. LAG-aging reaction using **BrPhCH$_2$** as template

LAG-aging experiment was performed using nanoparticulate zinc oxide (15 mg, mmol), imidazole (24 mg, mmol), **BrPhCH$_2$** (99 mg, mmol), and DEF (56 μL). The mixture was washed after 7 days, giving 0.6**BrPhCH$_2$**@RHO-Zn$_{16}$**Im**$_{32}$, as confirmed by NMR and PXRD (Figure S28, S27).

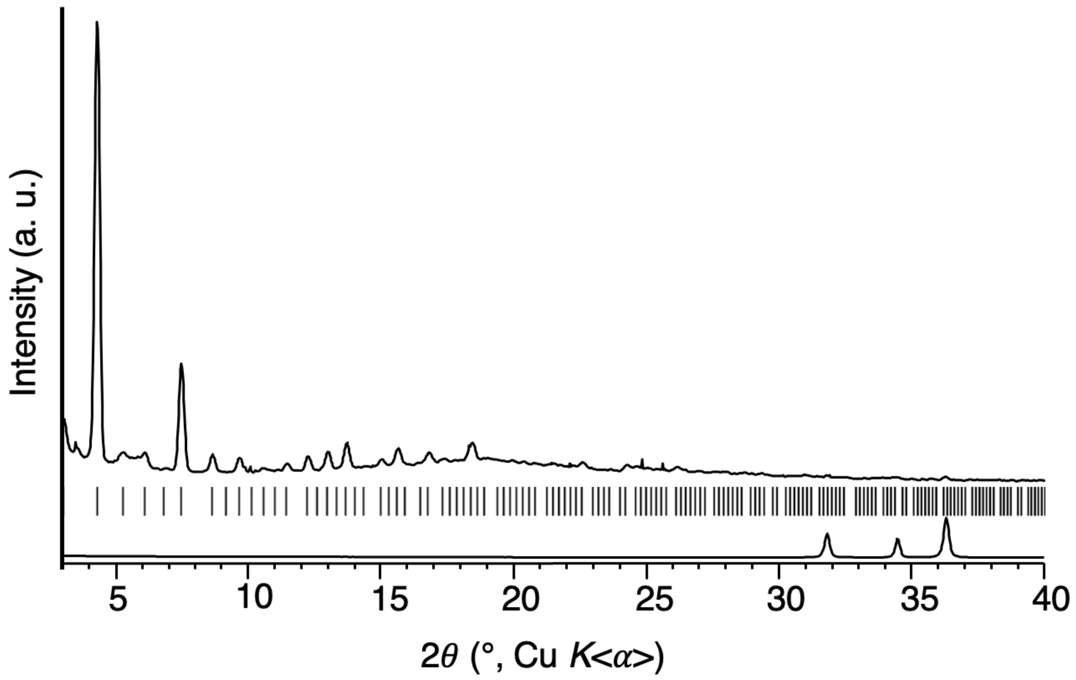

Figure S27. PXRD patterns of 0.6**BrPhCH$_2$**@RHO-Zn$_{16}$**Im**$_{32}$, synthesized by the LAG-aging procedure using **BrPhCH$_2$** and washed after 7 days (top), and zinc oxide (bottom). Tick marks denote predicted peak positions of a cubic unit cell, *Pm*-3*m*, *a* = 28.907(2) Å, corresponding to *x***MeMeCH$_2$**@RHO-Zn$_{16}$**Im**$_{32}$.

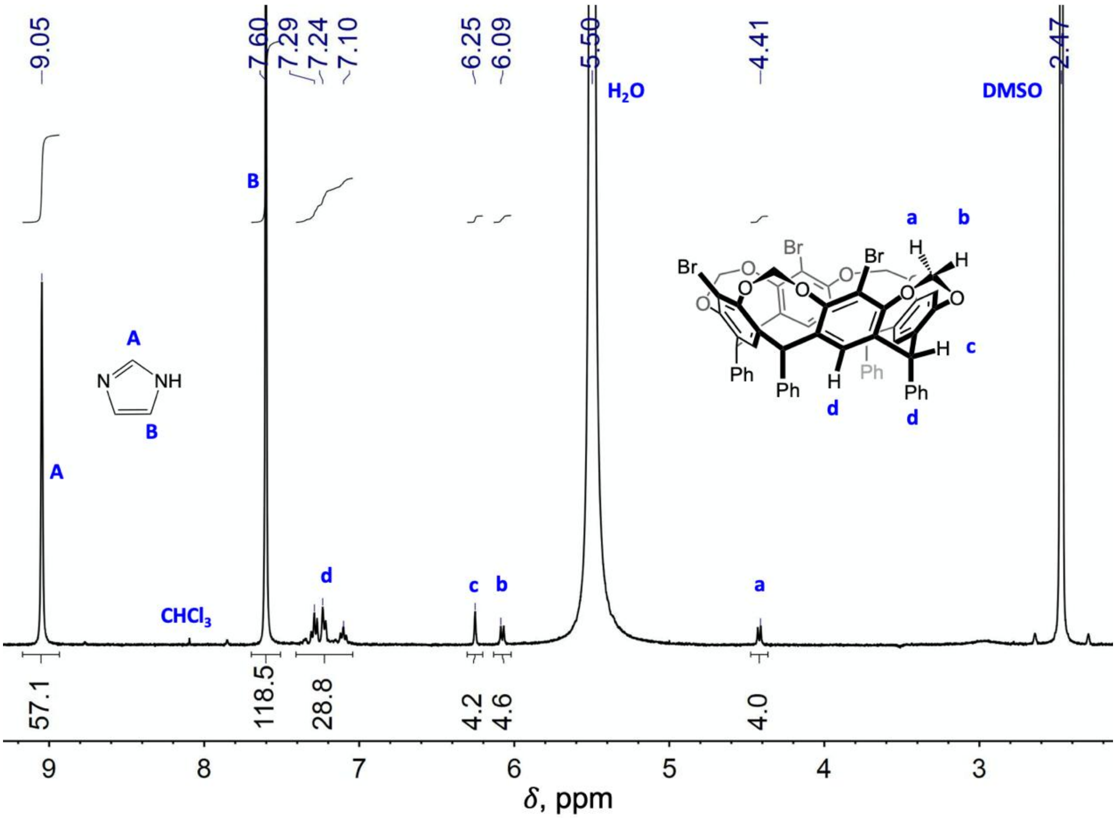

Figure S28. ^{1}H NMR spectrum (1 mL DMSO-d_6, 50μL DCl) of 0.6**BrPhCH**$_2$@RHO-Zn$_{16}$**Im**$_{32}$, synthesized by the LAG-aging procedure using **BrPhCH**$_2$ and washed after 7 days.

4. REFERENCES

1. Cram, D. J.; Stewart, K. D.; Goldberg, I.; Trueblood, K. N. Host-Guest Complexation. 49. Cavitands Containing Two Binding Cavities. *J. Am. Chem. Soc.* **1989**, *111*, 10.

2. Naumann, C.; Román, E.; Peinador, C.; Ren, T.; Patrick, B. O.; Kaifer, A. E.; Sherman, J. C. Expanding Cavitand Chemistry: The Preparation and Characterization of [n]Cavitands with n ≥ 4. *Chem. Eur. J.* **2001**, *7*, 1637–1645. DOI: 10.1002/1521-3765(20010417)7:8<1637::AID-CHEM16370>3.0.CO;2-X.

3. Kane, C. M.; Banisafar, A.; Dougherty, T. P.; Barbour, L. J.; Holman, K. T. Enclathration and Confinement of Small Gases by the Intrinsically 0D Porous Molecular Solid, Me,H,SiMe$_2$. *J. Am. Chem. Soc.* **2016**, *138*, 4377–4392. DOI: 10.1021/jacs.5b11395.

4. Cram, D. J.; Karbach, S.; Kim, H. E.; Knobler, C. B.; Maverick, E. F.; Ericson, J. L.; Helgeson, R. C. Host-Guest Complexation. 46. Cavitands as Open Molecular Vessels Form Solvates. *J. Am. Chem. Soc.* **1988**, *110*, 2229–2237. DOI: 10.1021/ja00215a037.

5. Kane, C. M.; Ugono, O.; Barbour, L. J.; Holman, K. T. Many Simple Molecular Cavitands Are Intrinsically Porous (Zero-Dimensional Pore) Materials. *Chem. Mater.* **2015**, *27*, 7337–7354. DOI: 10.1021/acs.chemmater.5b02972.

6. Kane, C. M. Crystalline Organic Cavitands As Microcavity Materials, Georgetown University, 2015.

7. Bryant, J. A.; Blanda, M. T.; Vincenti, M.; Cram, D. J.; Donegani, G.; Fauser, V. G. Guest Capture during Shell Closure. *J. Am. Chem. Soc.* **1991**, *113*, 2167–2172.

8. MestRe Nova, version 8.1.4-12489; Mestrelab Research S. L.: Santiago de Compostela, Spain, 2013. Mnova.

9. Degen, T. .; Sadki, M. .; Bron, E. .; König, U. .; Nénert, G. The HighScore Suite. *Powder Diffr.* **2014**, *29*, S13–S18.

10. Kourkoumelis, N.; O'Neill, L. ICDD Annual Spring Meetings: PowDLL, a Reusable .NET Component for Interconverting Powder Diffraction Data: Recent Developments. In *Powder Diffr.*; 2013; Vol. 28, pp 137–148.

11. CCDC. Mercury CSD. CCDC: Cambridge, UK 2019.

12. Yvon, K.; Jeitschko, W.; Parthé, E. LAZY PULVERIX, a Computer Program, for Calculating X-Ray and Neutron Diffraction Powder Patterns. *J. Appl. Cryst.* **1977**, *10*, 73–74.

13. Barbour, L. J. X-Seed - A Software Tool for Supramolecular Crystallography. *J. Supramol. Chem.* **2001**, *1*, 189–191.

14. Park, K. S.; Ni, Z.; Côté, A. P.; Choi, J. Y.; Huang, R.; Uribe-Romo, F. J.; Chae, H. K.; O'Keeffe, M.; Yaghi, O. M. Exceptional Chemical and Thermal Stability of Zeolitic Imidazolate Frameworks. *Proc. Natl. Acad. Sci. U. S. A.* **2006**, *103*, 10186–10191. DOI: 10.1073/pnas.0602439103.

15. He, M.; Yao, J.; Liu, Q.; Zhong, Z.; Wang, H. Toluene-Assisted Synthesis of RHO-Type Zeolitic Imidazolate Frameworks: Synthesis and Formation Mechanism of ZIF-11 and ZIF-12. *Dalt. Trans.* **2013**, *42*, 16608–16613. DOI: 10.1039/c3dt52103f.
